# Supplementary figures and images for: Pellioditis pelhamensis n. sp. (Nematoda: Rhabditidae) and Pellioditis pellio (Schneider, 1866), earthworm associates from different subclades within Pellioditis (syn. Phasmarhabditis Andrássy, 1976)
Source: PLoS One. 2023 Sep 6;18(9):e0288196. doi: 10.1371/journal.pone.0288196 (PMC10482300; doi:10.1371/journal.pone.0288196)

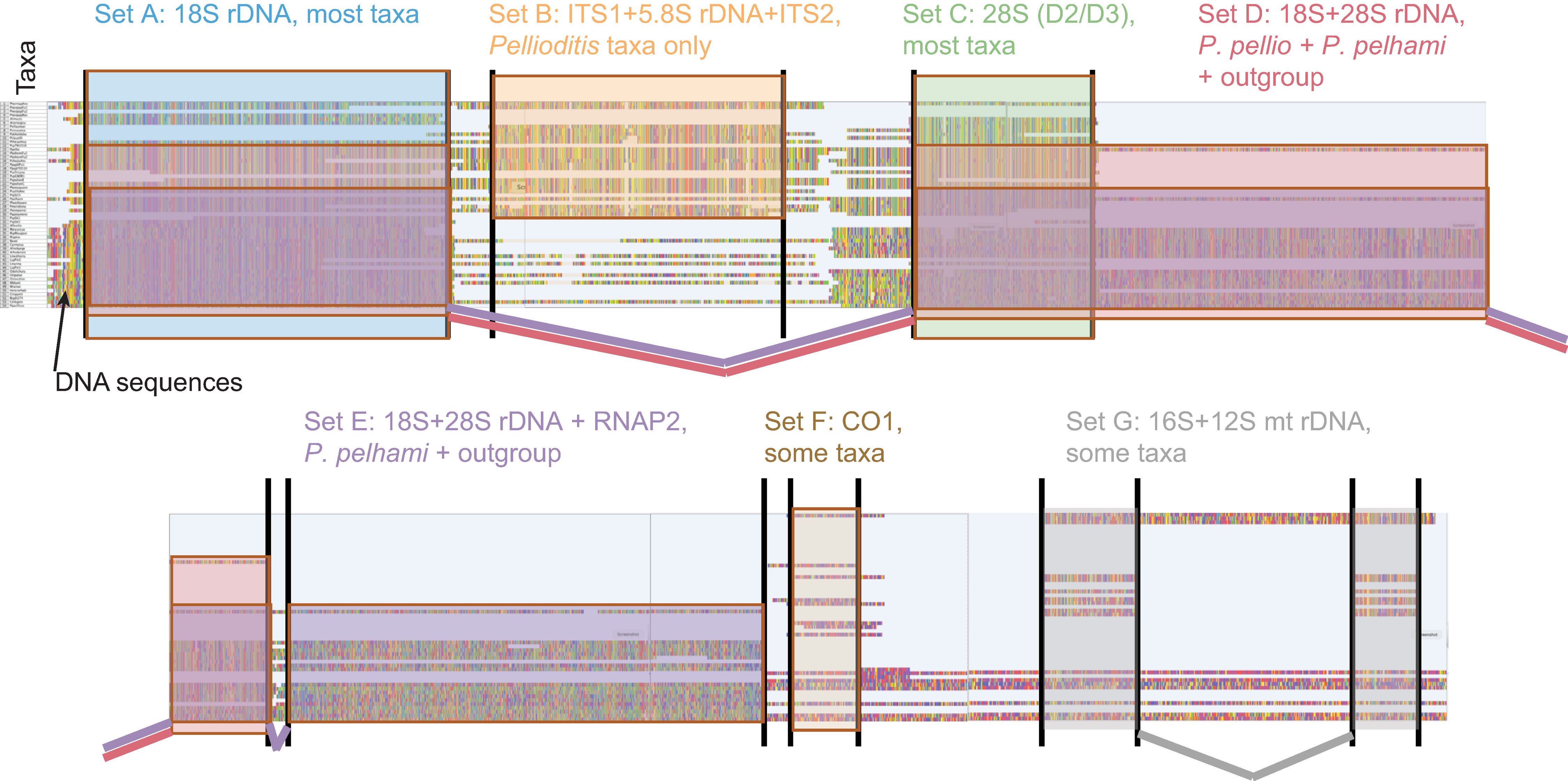

Supplement: S1 Fig — To conserve space, only the parsimony-informative characters (alignment positions) are depicted, with different colors depicting different nucleotide character states. Labeled boxes of different colors represent the taxon and character subsets of the full supermatrix used for individual ML bootstrap analyses. See Materials and Methods for description of datasets. (TIF) [file pone.0288196.s002.tif]
