# Supplementary material for: Pellioditis pelhamensis n. sp. (Nematoda: Rhabditidae) and Pellioditis pellio (Schneider, 1866), earthworm associates from different subclades within Pellioditis (syn. Phasmarhabditis Andrássy, 1976)
Source: PLoS One. 2023 Sep 6;18(9):e0288196. doi: 10.1371/journal.pone.0288196 (PMC10482300; doi:10.1371/journal.pone.0288196)
Supplement: S1 File — (DOCX) [file pone.0288196.s003.docx]

**S1 File. History of the *Pellioditis* and *Phasmarhabditis* taxa, and how the confusion between them arose.**

To more fully understand the sources of the taxonomic and nomenclatural confusion surrounding *Phasmarhabditis* and *Pellioditis* that our paper resolves, we relate here the history involved in the inception of these taxa and subsequent nomenclatural acts.

In his systematization of *Rhabditis sensu lato*, Osche [1] placed 16 species in the *Pellio* species group of *Rhabditis (Choriorhabditis)*, among them *R. caussaneli* Maupas, 1899, *R. mairei* Maupas, 1919, *R. neopapillosa* Mengert in Osche, 1952, *R. papillosa* (Schneider, 1866) and *R. pellio* (Schneider, 1866). He did not mention *R. hermaphrodita* (Schneider, 1859) or *R. foecunda* (Schneider, 1866); we now consider *R. caussaneli* and *R. foecunda* to be synonymous to *P. hermaphrodita* (which has priority). Dougherty [2] raised the *Pellio* species group to the subgenus level (under genus *Rhabditis*), giving the name *Pellioditis* to this subgenus and designating *P. pellio* as its type species. By 1955, Dougherty had included 19 species in this *Pellioditis* subgenus [3]. In his revision of Rhabditidae in 1976, Sudhaus [4] set up the *Papillosa* species group within *Pellioditis* (which he still regarded as a subgenus of genus *Rhabditis*) comprising seven species: *R. (P.) fruticicolae* Kreis, 1967, *R. (P.) hermaphrodita*, *R. (P.) incilaria* Yokoo & Shinohara, 1958, *R. (P.) mairei*, *R. (P.) neopapillosa*, *R. (P.) papillosa* (syn. *R. (P.) ninomiyai* Yokoo, 1968), and *R. (P.) pellio* (for English translation and update, see [5]). This *Papillosa* group would eventually become what we recognize today as genus *Pellioditis*.

*Pellioditis* was used for the first time as a genus-level name by Timm [6], apparently mistaken as a nomenclatural act by Andrássy [7, 8]. Note that ICZN Article 43, the Principle of Coordination, ensures that names, types and priority dates are all maintained automatically whenever genus/subgenus names change taxonomic level [9]. Thus, when Sudhaus formally raised *Pellioditis* to genus level in 2011 [10], it still retained its name and priority date—*Pellioditis* Dougherty, 1953—as well as its type species, *P. pellio*.

Andrássy [11] erected the new genus *Phasmarhabditis* in 1976, designating *P. papillosa* as its type species. Without addressing the suggestion by Sudhaus [4] that *P. pellio* and *P. papillosa* are members of the same clade, Andrássy [7, 8] maintained *Phasmarhabditis* and *Pellioditis* as separate taxa. In addition to *P. papillosa*, the type species of *Phasmarhabditis*, Andrássy moved *P. hermaphrodita*, *P. neopapillosa*, *P. nidrosiensis* (Allgén, 1933) and *P. valida* (Sudhaus, 1974) into *Phasmarhabditis*, while he placed *R. fruticicolae* and *R. incilaria* in *Pelodera* and kept *P. mairei*, *P. pellio* and *P. ninomiyai* in *Pellioditis*. The only change in the composition of *Phasmarhabditis* made later by Andrássy was to include *P. mairei* and to mistakenly list *P. valida* twice, as both a member of *Phasmarhabditis* and of *Pellioditis* [12]. Thus, by erecting *Phasmarhabditis*, Andrássy separated *P. papillosa* and *P. pellio* into two different genera. Since then, researchers describing new *Phasmarhabditis* species have not compared their finds with *P. pellio* or other *Pellioditis* species, presumably because the genus name limited their focus on which species were "comparable".

In contrast, beginning with Schneider ([13]: see here Fig. 9), all early authors emphasized the strong similarity between *P. papillosa* and *P. pellio*, differing only in female tail shape (dome-shaped versus conoid, respectively). Why Andrássy [7, 8, 12] ignored this, remains unknown, especially since he first correctly grouped the sister species *P. pellio* and *P. mairei* together in *Pellioditis* [7, 8], only later separating them into different genera [12].

Because it was clear from shared morphological characters that *P. papillosa* and *P. pellio* actually belong together in the same clade, Sudhaus treated *Phasmarhabditis* as a junior synonym of *Pellioditis* [10]. *Pellioditis* was the senior synonym and had priority because it was fixed much earlier as a genus-group name (1953 vs. 1976; see ICZN Article 23, Principle of Priority). (Sudhaus [10] also transferred *Rhabditis nidrosiensis* and *R. valida* to a different genus, *Buetschlinema*). In his compendium, Sudhaus [4] listed seven valid *Pellioditis* species (*P. fruticicolae* was judged to be synonymous to *P. incilaria*).

Starting with Wilson et al. [14] in the 1990s, *P. hermaphrodita* advanced to a biological control agent against slugs, and subsequent authors used the name *Phasmarhabditis* for this species. This might have been the reason why this genus name was perpetuated when, from 2015 until now, 14 closely related species were described as new. The name *Phasmarhabditis* was treated as valid "for the time being" [15], or was used "so as to avoid taxonomic uncertainty" [16] or "to avoid taxonomic confusion" [17, 18] while nevertheless accepting the priority of *Pellioditis* over *Phasmarhabditis*. According to a different argument [19-21], the synonymy of *Pellioditis* and *Phasmarhabditis* was "rejected" by mistakingly taking two outside-branching *Litoditis* species as alleged representatives of *Pellioditis* while ignoring *P. pellio* as a *bona fide* representative of *Pellioditis*. This error was likely due to the fact that GenBank was still using old names for the species taxa associated with DNA sequences used in these papers. These GenBank entries have since been updated. (This is a good example to highlight that GenBank is not a taxonomic authority.)

The current paper, along with rules established by the ICZN [9], resolves this nomenclatural controversy: (1) *Pellioditis pellio* is shown to be phylogenetically situated within the middle of the *"Phasmarhabditis"* clade (this paper, Fig. 10A), (2) *Pellioditis* has priority over *Phasmarhabditis* because it was established earlier as a genus name that is now applied to the same group of species (Article 23.1 Principle of Priority, and Article 23.3 regarding its application to synonymy; ICZN), and (3) as *P. pellio* is the type species of *Pellioditis*, it lends its associated genus name to the group (Article 61.1.1,Principle of Typification; ICZN). Thus, *Pellioditis* replaces *Phasmarhabditis* as the genus name, and *Phasmarhabditis* must be retired. We argue that this retirement of *Phasmarhabditis* will result in much less confusion. Furthermore, using "*Pellioditis*" for all these species will encourage researchers to consider comparing any new species to *all* closely related species, including *P. pellio*, avoiding further species synonymizations.

_____________________________

1. Osche G. Systematik und Phylogenie der Gattung *Rhabditis* (Nematoda). Zoologische Jahrbücher (Systematik); Zool Jb (Syst). 1952;81(3):190-280.

2. Dougherty EC. The genera of the subfamily Rhabditinae Micoletzky, 1922 (Nematoda). In: Dayal JS, Kunwar Suresh, editor. Thapar Commemoration Volume 1953 A Collection of Articles Presented to Prof G S Thapar on his Sixtieth Birthday. Lucknow, Uttar Pradesh, India: Department of Zoology, University of Lucknow; 1953. p. 69-76.

3. Dougherty EC. The genera and species of the subfamily Rhabditinae Micoletzky, 1922 (Nematoda): a nomenclatorial analysis—including an addendum on the composition of the family Rhabditidae Örley, 1880. Journal of Helminthology. 1955;29(3):105-52. Epub 5 June 2009. doi: 10.1017/S0022149X00024317.

4. Sudhaus W. Vergleichende Untersuchungen zur Phylogenie, Systematik, Ökologie, Biologie und Ethologie der Rhabditidae (Nematoda). Zoologica. 1976;43(125):1-229.

5. Sudhaus W, Fitch DHA. Comparative studies on the phylogeny and systematics of the Rhabditidae (Nematoda). Journal of Nematology. 2001;33(1):1-70. PubMed PMID: 19265873; PubMed Central PMCID: PMC2620500.

6. Timm RW. The widespread occurrence of the hemizonid. Nematologica. 1960;5(2):150. Epub Jan 1, 1960. doi: 10.1163/187529260X00523. PubMed PMID: 19319365; PubMed Central PMCID: PMC2620041.

7. Andrássy I. A Taxonomic Review of the Suborder Rhabditina (Nematoda: Secernentia). Paris, France: ORSTOM (Office de la Recherche Scientifique et Technique Outre-Mer), now IRD (Institut de Recherche pour le Développement); 1983. 241 p.

8. Andrássy I. Klasse Nematoda (Ordnungen Monhysterida, Desmoscolecida, Araeolaimida, Chromadorida, Rhabditida). Franz H, editor. Stuttgart, Germany: Gustav Fischer Verlag; 1984 1984. 509 p.

9. ICZN. International Code of Zoological Nomenclature. 4th ed. ed. London, UK: The International Trust for Zoological Nomenclature; 1999. 306 p.

10. Sudhaus W. Phylogenetic systematisation and catalogue of paraphyletic "Rhabditidae" (Secernentea, Nematoda). Journal of Nematode Morphology and Systematics. 2011;14(2):113-78.

11. Andrássy I. Evolution as a Basis for the Systematization of Nematodes. London: Pitman; 1976. 288 p.

12. Andrássy I. Free-living nematodes of Hungary (Nematoda errantia), I. Mahunka CCaS, editor. Budapest: Hungarian Natural History Museum and Systematic Zoology Research Group of the Hungarian Academy of Sciences; 2005. 518 p.

13. Schneider A. Monographie der Nematoden. Berlin, Germany: Georg Reimer; 1866. 357 p.

14. Wilson MJ, Glen DM, George SK. The rhabditid nematode *Phasmarhabditis hermaphrodita* as a potential biological-control agent for slugs. Biocontrol Sci Techn. 1993;3(4):503-11. Epub Sep. 17, 2008. doi: 10.1080/09583159309355306.

15. Tandingan De Ley I, Holovachov O, Mc Donnell RJ, Bert W, Paine TD, De Ley P. Description of *Phasmarhabditis californica* n. sp. and first report of *P. papillosa* (Nematoda: Rhabditidae) from invasive slugs in the USA. Nematology. 2016;18(2):175-93. Epub Mar. 18, 2016. doi: 10.1163/15685411-00002952.

16. Ross JL, Pieterse A, Malan AP, Ivanova E. *Phasmarhabditis safricana* n. sp. (Nematoda: Rhabditidae), a parasite of the slug *Deroceras reticulatum* from South Africa. Zootaxa. 2018;4420(3):391-404. Epub 2018/10/14. doi: 10.11646/zootaxa.4420.3.5. PubMed PMID: 30313534.

17. Huang RE, Ye W, Ren X, Zhao Z. Morphological and molecular characterization of *Phasmarhabditis huizhouensis* sp. nov. (Nematoda: Rhabditidae), a new rhabditid nematode from South China. PLoS One. 2015;10(12):e0144386. Epub 2015/12/18. doi: 10.1371/journal.pone.0144386. PubMed PMID: 26674768; PubMed Central PMCID: PMC4686017.

18. Ivanova ES, Spiridonov SE. *Phasmarhabditis meridionalis* sp n. (Nematoda: Rhabditidae) from a land snail *Quantula striata* (Gastropoda: Dyakiidae) from southern Vietnam. Russ J Nematol. 2017;25(2):129-40.

19. Nermut’ J, Půža V, Mekete T, Mráček Z. *Phasmarhabditis bonaquaense* n. sp. (Nematoda: Rhabditidae), a new slug-parasitic nematode from the Czech Republic. Zootaxa. 2016;4179(3):530-46. Epub 2016/11/05. doi: 10.11646/zootaxa.4179.3.8. PubMed PMID: 27811685.

20. Nermut’ J, Půža V, Mekete T, Mráček Z. *Phasmarhabditis bohemica* n. sp. (Nematoda: Rhabditidae), a slug-parasitic nematode from the Czech Republic. Nematology. 2017;19(1):93-107. doi: 10.1163/15685411-00003034.

21. Nermut’ J, Půža V, Mráček Z. *Phasmarhabditis apuliae* n. sp. (Nematoda: Rhabditidae), a new rhabditid nematode from milacid slugs. Nematology. 2016;18:1095-112. doi: 10.1163/15685411-00003017.
