# Supplementary material for: Pellioditis pelhamensis n. sp. (Nematoda: Rhabditidae) and Pellioditis pellio (Schneider, 1866), earthworm associates from different subclades within Pellioditis (syn. Phasmarhabditis Andrássy, 1976)
Source: PLoS One. 2023 Sep 6;18(9):e0288196. doi: 10.1371/journal.pone.0288196 (PMC10482300; doi:10.1371/journal.pone.0288196)
Supplement: Attachment — Submitted filename: PONE-D-23-19233_reviewer (revised).pdf [file pone.0288196.s005.pdf]

PLOS ONE

<i>Pellioditis pelhami</i> n. sp. (Nematoda: Rhabditidae) and <i>Pellioditis pellio</i> (Schneider, 1866), earthworm associates from different subclades within <i>Pellioditis</i> (syn. <i>Phasmarhabditis</i> Andrássy, 1976)

--Manuscript Draft--

**Manuscript Number:**	PONE-D-23-19233
**Article Type:**	Research Article
**Full Title:**	<i>Pellioditis pelhami</i> n. sp. (Nematoda: Rhabditidae) and <i>Pellioditis pellio</i> (Schneider, 1866), earthworm associates from different subclades within <i>Pellioditis</i> (syn. <i>Phasmarhabditis</i> Andrássy, 1976)
**Short Title:**	<i>Pellioditis pelhami</i> n. sp. and <i>Pellioditis pellio</i>
**Corresponding Author:**	David H. A. Fitch<br>New York University<br>New York, NY UNITED STATES
**Keywords:**	slug parasites; nematodes; molecular phylogeny; earthworm necromeny; morphological description; SEM
**Abstract:**	Recently, much attention has been focused on a group of rhabditid nematodes called <i>Phasmarhabditis</i>, a junior synonym of <i>Pellioditis</i>, as a promising source of biocontrol agents for invasive slugs. <i>Pellioditis pelhami</i> n. sp. was first isolated from earthworms near Pelham Bay Park in Bronx, New York, USA, in 1990, and has been found to be pathogenic to slugs as well as some earthworms. It has also been used in several comparative developmental studies. Here, we provide a description of this species, as well as a redescription of a similar earthworm-associated nematode, <i>Pellioditis pellio</i> Schneider, 1866, re-isolated from the type locality. Although <i>P. pelhami</i> n. sp. and <i>P. pellio</i> are morphologically similar, they are reproductively isolated. Molecular phylogenetic analysis places both species in a clade that includes all species previously described as <i>Phasmarhabditis</i> which are associated with gastropods. <i>Phasmarhabditis</i> Andrássy, 1976 is therefore a junior synonym of <i>Pellioditis</i> Dougherty, 1953. Also, <i>Phasmarhabditis bohemica</i> Nermut', Půža, Mekete & Mráček, 2017, described to be a facultative parasite of slugs, is found to be a junior synonym of <i>Pellioditis pellio</i> (Schneider, 1866), adding to evidence that <i>P. pellio</i> is associated with both slugs and earthworms. The earthworm-associated species <i>P. pelhami</i> n. sp. and <i>P. pellio</i> represent different subclades within <i>Pellioditis</i>, suggesting that <i>Pellioditis</i> species in general have a broader host range than just slugs. Because of this, caution is warranted in using these species as biological control agents until more is understood about their ecology.
**Order of Authors:**	Irma Tandingan De Ley
	Karin Kiontke
	Wim Bert
	Walter Sudhaus
	David H. A. Fitch
**Additional Information:**	
**Question**	**Response**
**Financial Disclosure**<br><br>Enter a financial disclosure statement that describes the sources of funding for the work included in this submission. Review the submission guidelines for detailed requirements. View published research	This study was supported by National Institutes of Health (https://www.nih.gov) grant R01GM141395 to DHAF. The funders had no role in study design, data collection and analysis, decision to publish, or preparation of the manuscript.

Powered by Editorial Manager® and ProduXion Manager® from Aries Systems Corporation

articles from *PLOS ONE* for specific examples.<br><br>This statement is required for submission and **will appear in the published article** if the submission is accepted. Please make sure it is accurate.<br><br>**Unfunded studies**<br>Enter: *The author(s) received no specific funding for this work.*<br><br>**Funded studies**<br>Enter a statement with the following details:<br>• Initials of the authors who received each award<br>• Grant numbers awarded to each author<br>• The full name of each funder<br>• URL of each funder website<br>• Did the sponsors or funders play any role in the study design, data collection and analysis, decision to publish, or preparation of the manuscript?<br>• **NO** - Include this sentence at the end of your statement: *The funders had no role in study design, data collection and analysis, decision to publish, or preparation of the manuscript.*<br>• **YES** - Specify the role(s) played.<br><br>* typeset	
**Competing Interests**<br><br>Use the instructions below to enter a competing interest statement for this submission. On behalf of all authors, disclose any competing interests that could be perceived to bias this work—acknowledging all financial support and any other relevant financial or non-financial competing interests.<br><br>This statement is required for submission and **will appear in the published article** if the submission is accepted. Please make sure it is accurate and that any funding sources listed in your Funding Information later in the submission form are also declared in your Financial Disclosure statement.	The authors have declared that no competing interests exist.

View published research articles from *PLOS ONE* for specific examples.<br><br>**NO authors have competing interests**<br><br>Enter: *The authors have declared that no competing interests exist.*<br><br>**Authors with competing interests**<br><br>Enter competing interest details beginning with this statement:<br><br>*I have read the journal's policy and the authors of this manuscript have the following competing interests: [insert competing interests here]*<br><br>* typeset	
**Ethics Statement**<br><br>Enter an ethics statement for this submission. This statement is required if the study involved:<br><br>• Human participants<br>• Human specimens or tissue<br>• Vertebrate animals or cephalopods<br>• Vertebrate embryos or tissues<br>• Field research<br><br>Write "N/A" if the submission does not require an ethics statement.<br><br>General guidance is provided below. Consult the submission guidelines for detailed instructions. **Make sure that all information entered here is included in the Methods section of the manuscript.**	N/A

Powered by Editorial Manager® and ProduXion Manager® from Aries Systems Corporation

**Format for specific study types**<br><br>**Human Subject Research (involving human participants and/or tissue)**<br>• Give the name of the institutional review board or ethics committee that approved the study<br>• Include the approval number and/or a statement indicating approval of this research<br>• Indicate the form of consent obtained (written/oral) or the reason that consent was not obtained (e.g. the data were analyzed anonymously)<br><br>**Animal Research (involving vertebrate animals, embryos or tissues)**<br>• Provide the name of the Institutional Animal Care and Use Committee (IACUC) or other relevant ethics board that reviewed the study protocol, and indicate whether they approved this research or granted a formal waiver of ethical approval<br>• Include an approval number if one was obtained<br>• If the study involved *non-human primates*, add *additional details* about animal welfare and steps taken to ameliorate suffering<br>• If anesthesia, euthanasia, or any kind of animal sacrifice is part of the study, include briefly which substances and/or methods were applied<br><br>**Field Research**<br><br>Include the following details if this study involves the collection of plant, animal, or other materials from a natural setting:<br>• Field permit number<br>• Name of the institution or relevant body that granted permission	
**Data Availability**<br><br>Authors are required to make all data underlying the findings described fully available, without restriction, and from the time of publication. PLOS allows rare exceptions to address legal and ethical concerns. See the PLOS Data Policy and FAQ for detailed information.	Yes - all data are fully available without restriction

Powered by Editorial Manager® and ProduXion Manager® from Aries Systems Corporation

A Data Availability Statement describing where the data can be found is required at submission. Your answers to this question constitute the Data Availability Statement and **will be published in the article**, if accepted.<br><br>**Important:** Stating 'data available on request from the author' is not sufficient. If your data are only available upon request, select 'No' for the first question and explain your exceptional situation in the text box.<br><br>Do the authors confirm that all data underlying the findings described in their manuscript are fully available without restriction?	
**Describe where the data may be found in full sentences. If you are copying our sample text, replace any instances of XXX with the appropriate details.**<br><br>• If the data are **held or will be held in a public repository**, include URLs, accession numbers or DOIs. If this information will only be available after acceptance, indicate this by ticking the box below. For example: *All XXX files are available from the XXX database (accession number(s) XXX, XXX.*).<br>• If the data are all contained **within the manuscript and/or Supporting Information files**, enter the following: *All relevant data are within the manuscript and its Supporting Information files.*<br>• If neither of these applies but you are able to provide **details of access elsewhere**, with or without limitations, please do so. For example:<br><br>*Data cannot be shared publicly because of [XXX]. Data are available from the XXX Institutional Data Access / Ethics Committee (contact via XXX) for researchers who meet the criteria for access to confidential data.*<br><br>*The data underlying the results presented in the study are available from (include the name of the third party*	Sequence files are available from the GenBank database (accession numbers OR059186 and OR059187). All other data are within the manuscript and its Supporting Information files.

and contact information or URL).<br>• This text is appropriate if the data are owned by a third party and authors do not have permission to share the data.<br><br>* typeset	
Additional data availability information:	Tick here if the URLs/accession numbers/DOIs will be available only after acceptance of the manuscript for publication so that we can ensure their inclusion before publication.

Powered by Editorial Manager® and ProduXion Manager® from Aries Systems Corporation

Pellioditis pelhami n. sp. (Nematoda: Rhabditidae) and *Pellioditis pellio* (Schneider, 1866), earthworm associates from different subclades within *Pellioditis* (syn. *Phasmarhabditis* Andrássy, 1976)

Short title:

Pellioditis pelhami n. sp. and *Pellioditis pellio*

Irma Tandingan De Ley[1], Karin Kiontke[2], Wim Bert[3], Walter Sudhaus[4], and David H. A. Fitch*[2]

1 Department of Nematology, University of California, Riverside, CA, USA; ORCID 0000-0002-9399-0693

2 Department of Biology, New York University, New York 10003 USA;

3 Nematology Unit, Department of Biology, Ghent University; K.L. Ledeganckstraat 35, 9000 Ghent, Belgium; ORCID 0000-0002-5864-412X

4 Institut für Biologie/Zoologie, Freie Universität Berlin, Königin-Luise-Str. 1–3, 14195 Berlin, Germany

* Corresponding author: david.fitch@nyu.edu (DHAF), ORCID 0000-0001-6940-7544

Keywords:

slug parasites, nematodes, molecular phylogeny, earthworm necromeny, morphological description, SEM

Abstract

Recently, much attention has been focused on a group of rhabditid nematodes called *Phasmarhabditis*, a junior synonym of *Pellioditis*, as a promising source of biocontrol agents for invasive slugs. *Pellioditis pelhami* n. sp. was first isolated from earthworms near Pelham Bay Park in Bronx, New York, USA, in 1990 and has been found to be pathogenic to slugs as well as some earthworms. It has also been used in several comparative developmental studies. Here, we provide a description of this species, as well as a redescription of a similar earthworm-associated nematode, *Pellioditis pellio* Schneider, 1866, re-isolated from the type locality. Although *P. pelhami* n. sp. and *P. pellio* are morphologically similar, they are reproductively isolated. Molecular phylogenetic analysis places both species in a clade that includes all species previously described as *Phasmarhabditis* which are associated with gastropods. *Phasmarhabditis* Andrássy, 1976 is therefore a junior synonym of *Pellioditis* Dougherty, 1953. Also, *P. bohemica* Nermut', Půža, Mekete & Mráček, 2017, described to be a facultative parasite of slugs, is found to be a junior synonym of *Pellioditis pellio* (Schneider, 1866), adding to evidence that *P. pellio* is associated with both slugs and earthworms. The earthworm-associated species *P. pelhami* n. sp. and *P. pellio* represent different subclades within *Pellioditis*, suggesting that *Pellioditis* species in general have a broader host range than just slugs. Because of this, caution is warranted in using these species as biological control agents until more is understood about their ecology.

Introduction

Combatting pest organisms with biological means is an attractive alternative to pest control with chemicals. However, biological control is not without risks. In the past, several species meant to serve as specific biocontrol agents had unintended negative consequences for biodiversity (reviewed in [1]). This is especially true if a species used for biological control is not specific for the intended target, or if it colonizes habitats far outside of the range of the initial application.

The continued search for safer alternatives to toxic molluscicides in mitigating invasive gastropod pests led to the successful commercialization of the nematode *Phasmarhabditis hermaphrodita* (a.k.a. *Pellioditis hermaphrodita*) as the biological control agent Nemaslug®. This search also focused interest into related species. Of the 21 species described so far in this group (15 discovered since 2015), all but three were isolated from slugs or snails [2]. However, one species related to *P. hermaphrodita*, represented by strains EM434 and DF5056 and until now unnamed, was isolated from earthworms [3]. Two additional strains, DL308 and DL316 (with identical mitochondrial DNA sequences as EM434) were recently recovered in Oregon from the slugs *D. reticulatum* [4] and *Testacella* sp. (D. Howe & D. Denver, pers. comm.). Thus, this species naturally uses both molluscs and earthworms as natural hosts. Two other related species, *Pellioditis pellio* and *Pellioditis mairei*, were originally described as associates of earthworms, but *P. pellio* (described as *P. bohemica*, syn. nov.) has also been reported from slugs [5, 6]. Thus, at least some species in this group are not specific to mollusc hosts.

Recent experiments showed that EM434/DF5056 causes mortality to the agriculturally important slug pests *Deroceras reticulatum* and *Ambigolimax valentianus* and to the earthworm species *Eisenia hortensis* and *Eisenia fetida* (composting worms), but not to *Lumbricus* [7]. Thus, even though the natural association of these species with their host appears to be "necromenic", under experimental conditions or perhaps in a non-native host, they are pathogenic. Necromeny (“waiting for the cadaver”) describes a lifestyle in which nematode dauer larvae enter a host but do not resume development until after the host dies [8]. Subsequently, they exit the dauer stage and develop on the decaying cadaver. Necromenic

nematodes are normally not harmful to the host; e.g. earthworms may encapsulate them in “brown bodies” [9].

To avoid unintentional effects on the earthworm population, should further *Pellioditis* (syn. *Phasmarhabditis*) species be developed as biological control agents, the host range of these species needs to be more fully known. As a beginning, it is worthwhile to ask if the earthworm association is an evolutionary novelty within the group or an ancestral feature of the group. Knowledge of the phylogenetic relationships of the species in the taxon can help decide between these possibilities, but it requires molecular data for the earthworm-associated species, and thus isolation of a reliable strain of *P. pellio*.

Reconstructing the phylogeny of *Pellioditis* including *P. pellio* should also settle a taxonomic controversy involving the genus [2, 10]. In 2011, Sudhaus [11] declared *Phasmarhabditis* to be a junior synonym of *Pellioditis.* This was because *P. pellio,* the type species of *Pellioditis,* was recognized as part of the taxon based on morphological similarities. Once a type species is transferred into a different genus (here into *Phasmarhabditis*), its genus name (here *Pellioditis)* has priority if it is an older name; the junior synonym should then be retired (see S1 File; ICZN Articles 23 and 61 [12]). This synonymization was not generally accepted by researchers working on mollusc-pathogenic nematodes who continued to use the name *Phasmarhabditis*. They argued either that the preferred genus name was a matter of "diverse perspectives of revisions" [13, 14], or that *Phasmarhabditis* and *Pellioditis* were "clearly separated phylogenetically" [6, 15, 16]. The latter argument was derived from a mistaken taxon sampling in molecular phylogenetic analyses, where the genus *Pellioditis* was represented by two species, *Litoditis marina* and *L. mediterranea,* that were listed as *Pellioditis* species in GenBank (now corrected). These species had been removed earlier from *Pellioditis* [11] and transferred into genus *Litoditis* after a molecular phylogenetic analysis of rhabditid nematodes revealed that the genus was previously polyphyletic [17]. Because this analysis did not include *P. pellio*, and indeed, no sequences for *P. pellio* were yet available in databases, *Phasmarhabditis* researchers might be forgiven for their confusion over the taxonomy (see S1 File for a full history of *Pellioditis* and *Phasmarhabditis* taxonomy and arguments for synonymy).

Here, we provide a description of the earthworm-associated species represented by strain EM434

as *Pellioditis pelhami* n. sp. and a redescription of *P. pellio* based on a new isolate collected from the type location and type host. We reconstructed a phylogeny using molecular sequence data we obtained for *P. pellio* and *P. pelhami* along with all available sequences of species described as *Phasmarhabditis* as well as representatives of the outgroup and of the two mollusc-parasitic clades *Agfa* and Angiostomatidae. This analysis shows clearly that *P. pellio* is part of a monophyletic clade that includes all *Pellioditis/Phasmarhabditis* species plus Angiostimatidae. *P. pelhami* and *P. pellio* belong to two different subclades of *Pellioditis,* indicating that earthworms were either acquired as hosts twice independently, or that an earthworm association is an ancestral feature of *Pellioditis.*

Materials and methods

Nematode isolation and culture

Strains EM434 and DF5056 were isolated from earthworms, which were first washed in tap water and then chopped up on a nematode growth medium (NGM) agar plate. Rhabditids that came out were collected over the next few days. One individual gravid female was picked to start each strain. Cultures were maintained on *Escherichia coli* (OP50). Strains were cryogenically maintained shortly after recovery. EM434 was used for the description reported here.

P. pellio strain SB361 was isolated by WS from the type location for this species [18]. Pieces of earthworm were placed directly on water agar and emerging nematodes were recovered. A culture was provided to the Fitch lab and cryogenically preserved on June 17, 2005, by KK. SB361 was thawed in 2022, grown on NGM with *E. coli*, and used for the redescription reported here.

Identification of nematodes

That strains EM434 and DF5056 belong to the same species was shown by interfertility and essentially identical nucleotide sequences for SSU and LSU (small and large subunit ribosomal RNA genes) and CO1

(cytochrome oxidase I gene) [7]. Detailed morphological characterization was thus performed on only one strain, EM434, as the representative strain for the species. EM434 was previously suspected to be *P. pellio* based on morphology, so rRNA (ribosomal RNA) genes including the ITS (internal transcribed spacer) regions were sequenced from strains SB361 and EM434 (see S1 Table). Ribosomal RNA gene sequences have proven to be good for phylogenetic analysis of rhabditids and the ITS sequences for barcodes [17, 19-23].

Measurements were obtained from nematodes fixed in double-strength formalin-glycerol and processed to anhydrous glycerin for light microscopy as previously described [24, 25]. Some measurements were also obtained from live nematodes anaesthetized with sodium azide and not found to be significantly different (data not shown). Line drawings were made from live, anaesthetized nematodes using a Zeiss Axioplan microscope equipped with a drawing tube and DIC (differential interference contrast) optics.

Scanning electron microscopy (SEM)

SEM was performed at the University of Ghent, Belgium as described previously [26] with minor modifications. Specifically, nematodes were fixed in 4:1 formalin-glycerol, rinsed three times with PBS at 10 minute-intervals, followed by another two rinses of distilled water at 10 minute intervals and sonication for 8 min. Nematodes, in as little liquid as possible, were placed inside a metal cylinder lined with fine mesh. This was followed by a dehydration series in increasing concentrations of ethanol, starting at 30% for 15 min., transferred successively to 50, 65, 75, 85, 95 and 98% each for 20 min. and three times at 100% for 10 min. Dehydrated nematodes were critical-point-dried using a Balzers Union dryer (CPD020) mounted on the surface of double-sided conductive carbon tape on a stub, sputter-coated with 25 nm layer gold (Jeol, SOP Renaat Dasseville) for 3 min., and observed on a JEOL JSM-840 scanning electron microscope at 5 kV.

Immunofluorescent staining

Adherens junctions in EM434 were stained using MH27 monoclonal antibody as detailed previously [27].

The patterns of junctions in the mid-J4 males were used to assign homologies of ray (genital papilla, GP) cells with those of other rhabditids [23, 27, 28] using GP nomenclature described previously [29].

Molecular phylogenetic analyses

DNA sequencing of the entire ribosomal RNA (rRNA) gene repeat unit (18S or SSU rRNA, 28S or LSU rRNA, the 5.8S rRNA gene and flanking internal transcribed spacers ITS1 and ITS2) was performed as previously described [17]. GenBank accession numbers are OR059186 (nearly complete ribosomal RNA gene repeat unit for *P. pelhami* n. sp. strain EM434) and OR059187 (nearly complete ribosomal RNA gene repeat unit for *P. pellio* strain SB361).

Corresponding sequences of representative rhabditid nematode taxa were downloaded from GenBank (S1 Table) and preliminary alignments were obtained using Clustal Omega [30] with default parameters. A supermatrix was constructed by concatenating the alignments of the nuclear SSU, ITS1, 5.8S, ITS2 and LSU rRNA genes, RNAP2 (RNA polymerase II large subunit) coding regions (introns were deleted), and mitochondrial CO1, 16S and 12S rRNA genes, with flanking portions of the mitochondrial genomes. Where available, nuclear and mitochondrial genome sequences were used to scaffold the supermatrix. Manual alignment using Mesquite (ver. 4.70 build 940) [31] was used to optimize the alignment in several regions. For example, for protein-coding loci, inferred amino acid sequences were used with the appropriate nuclear or mitochondrial genetic code to align the cognate nucleotide sequences. Also, the reverse complement of several of the sequences had to be used, since GenBank accessions for these sequences are not uniform with respect to sequence orientation (see S1 Table). BBEdit (ver. 14.6.4) was also used for editing of sequences, alignments and NEXUS commands.

A major problem was that each species is represented by multiple, often different and noncontiguous or nonoverlapping fragments of DNA sequences. Having each sequence represent a separate operational taxonomic unit (OTU) generates a very sparse data matrix with many OTUs and a correspondingly enormous treespace that would be difficult to sample for heuristic searches. We thus

decided to reduce the number of OTUs while maintaining as much sequence coverage as possible for each species taxon. To do this, we collapsed several sequences representing the same species into a single OTU by (1) concatenating sequences for different genes from the same species into a supermatrix, even if they derived from different strains, and (2) reconstructing a most parsimonious ancestral sequence for four cases in which the same gene was represented by multiple sequences from different strains/isolates: *P. hermaphrodita*, *P. zhejiangensis*, *P. papillosa* (keeping separate strain ITD510), and *P. californica* (see S1 Table). The ancestral reconstructions did not involve many sites and was performed manually using other closely related species as outgroup representatives; conventional ambiguity codes were used where a sequence or ancestral state could not be unambiguously determined. Although this produced a supermatrix with a single OTU for most species, four species were still represented by 2-3 OTUs: *P. bohemica*, *P. apuliae*, *P. papillosa* and *P. neopapillosa*. The final supermatrix comprised 13,522 nucleotide characters (alignment positions) and 53 OTUs representing 48 species (NEXUS file available from DHAF on request).

To test robustness to phylogenetic inference method, the supermatrix was analyzed using weighted parsimony (WP) and maximum likelihood (ML) approaches. For WP, the supermatrix was reduced to only the phylogenetically-informative characters (4,578 nucleotide characters) for the 48 species (53 OTUs). Because parsimony does not require an evolutionary model, it is predicted to be quite robust to missing data given a fair representation of characters [32]. WP analysis was performed with PAUP* [33] using a stepmatrix that weighted transversions twice that of transitions, i.e. "weighted parsimony", demonstrated to be significantly more accurate than unweighted parsimony [34]. Several heuristic searches were conducted, using random addition of sequences to get a starting tree (addseq=random), with 1000 replications (nreps=1000) and the default TBR search method. In each of three separate searches, the same set of 27 equally most-parsimonious (MP) trees were found. In the largest search, approximately $4X10^8$ trees were evaluated. To assess the robustness of the data with respect to phylogenetic bipartitions, jackknife analysis was performed (100 replicates with 25% random character deletion, each replicate employing a heuristic search with the following parameters: addseq=random, swap=tbr, nreps=10).

For ML, a supertrees approach was used. In this case, a complete set of characters is needed per

character set (e.g. gene) to ensure appropriate evolutionary model and parameter estimation. Because the supermatrix was somewhat sparse, the matrix was divided into seven subsets of characters and taxa, minimizing the amount of missing data for each subset (S1 Fig). DatasetA covered a part of the SSU gene and comprised 1,612 characters for 41 OTUs; DatasetB covered ITS and 5.8S genes (717 characters) only for 18 OTUs in the *Pellioditis* clade; DatasetC included the D2/D3 region of the LSU gene (901 characters) for 42 OTUs; DatasetD consisted of nearly complete SSU and LSU sequences (4,963 characters) for two of the *Pellioditis* species (*P. pellio* and *P. pelhami* n. sp.) plus 18 outgroup rhabditids; DatasetE included nearly complete SSU, LSU and RNAP2 sequences (6,881 characters) for *P. pelhami* n. sp. and 17 outgroup rhabditids; DatasetF only included partial sequences from the CO1 gene (474 characters) for 8 *Pellioditis* OTUs plus 7 outgroup rhabditids; and DatasetG covered partial sequences from the mitochondrial 12S and 16S rRNA genes (1,176 characters) for 10 *Pellioditis* OTUs and 7 outgroup rhabditids. RAxML [35] was used to perform ML bootstrapping analysis individually on each of the seven subsets (1000 bootstrap replications using a GTR substitution model with a gamma correction for rate variation and invariant sites, with parameters estimated from the data). Nodes (bipartitions) supported ≤60% in bootstrap analyses were collapsed and the resulting trees were used in a supertree analysis using Clann ver. 4.2.5 [36]. Using the MPR (matrix representation using parsimony) criterion, a heuristic search (parameters: TBR branch-swapping, addseq=simple, swap=tbr, nreps=100) of >$3X10^6$ supertrees yielded 133,302 MPR trees for which a majority-rule consensus was constructed; nodes represented in ≤70% of the supertrees were collapsed to represent uncertainty.

Nomenclatural acts

The electronic edition of this article conforms to the requirements of the amended International Code of Zoological Nomenclature (ICZN), and hence the new names contained herein are available under that Code from the electronic edition of this article. This published work and the nomenclatural acts it contains have been registered in ZooBank, the online registration system for the ICZN. The ZooBank LSIDs (Life Science

Identifiers) can be resolved and the associated information viewed through any standard web browser by appending the LSID to the prefix “http://zoobank.org/”. The LSIDs for this publication are: urn:lsid:zoobank.org:act:C7EC39A9-3D17-47E8-B9FB-AF676B0DDF29 and urn:lsid:zoobank.org:act:C1CDBEEF-A1BB-4B0A-AF6A-BC8051416FD8. The electronic edition of this work was published in a journal with an ISSN, and has been archived and is available from the following digital repositories: LOCKSS.

Results and Discussion

Pellioditis pelhami Tandingan De Ley, Kiontke, Bert, Sudhaus & Fitch n. sp.

ZooBank LSID: urn:lsid:zoobank.org:act:C7EC39A9-3D17-47E8-B9FB-AF676B0DDF29

Strains:

- EM434: leg. S. E. Baird, 1990, from an unidentified earthworm, Bronx, New York, USA (40.851009, -73.846283). Synonymous designations of this strain:
  = *Pellioditis* sp. [3]
  = *Pellioditis* sp. DF5039 [37]
  = *Pellioditis* sp. EM434 [38-42]
  = *Phasmarhabditis* sp. EM434 [6, 13, 15-17, 26, 43-54]
  = *Phasmarhabditis* sp. [55, 56], using sequence accession numbers EU195967 and EU196008.
- DF5056: leg. D. Fitch, 1993, Bronx, New York, USA (40.852514, -73.788823) from an unidentified earthworm. Nuclear and mitochondrial ribosomal RNA gene sequences identical to those of EM434; interfertile with EM434 [7].
- DL308: from a gray garden slug, *D. reticulatum*, Oregon, USA. Nuclear and mitochondrial ribosomal RNA gene sequences identical to those of EM434 [4].

- DL316 from a shelled slug, *Testacella*, Oregon, USA. Molecular sequences identical to those of EM434 (Dana Howe & Dee Denver, pers. comm.).

Description

Based on strain EM434.

Illustrations. Figs 1–4, supplemental videos showing stacks of DIC focal planes through live animals (S1 Video–S9 Video).

Fig 1. Line drawings of *Pellioditis pelhami* n. sp., strain EM434. (A–E, G) Adult female: (A) anterior region , left side view, excretory pore (ep) at the level of the grinder. (B, C) Tail region: (B) left side view, (C) ventral view (compare with Fig 2F), showing prominent phasmids approximately halfway down the tail from the anus, protruding through the thick cuticle and terminating on or slightly outside of the external surface. (D) Anterior end showing stoma region, ventral view (compare with Fig 2B showing a left side view). (E) Metarhabdion showing tiny wart-like protrusions. (F) Stoma region of J2, left side view, showing that the stoma is relatively longer and narrower than in the adult. (G) Lateral field of adult female showing ridges (compare with Fig 2A showing same for male). (H–L) Male: ventral view (H) and left side view (I) of adult male tail with genital papillae (GPs) labeled according to the homology system developed for Rhabditina [28, 29] based on common developmental origins of the GPs in the lateral epidermis [23, 27, 57, 58]. Accordingly, there are two GPs—the anterior dorsal (ad) and posterior dorsal (pd)—with cell lineages originating more dorsally than the seven others (v1–v7) (compare Fig 1J). The small ray-like phasmids (ph) are only seen in ventral view. (J) Adherens junctions of the ray cell and lateral seam cells in the left lateral epidermis of a J4 male (anterior up, dorsal to the right) as visualized by immunostaining with MH27 monoclonal antibody at the time when external ray papillae are forming (compare Fig 2D). (K) Right spicule, left side view; notice the complex tip (compare with Fig 2G). (L) Gubernaculum, ventral view (compare Fig 2G).

Fig 2. DIC photomicrographs of *P. pelhami* n. sp. (A) Lateral field of an adult male. (B) Adult male head with stoma, sagittal plane, left side view. (C) Adult male pharynx region, left side view. (D) J4 male tail, left side view, showing developing genital papillae and phasmid (ph) as it is drawn out in the wake of tail tip morphogenesis in this peloderan species; ad and pd indicate the anterior dorsal and posterior dorsal genital papilla (compare Fig 1H–J). (E) Posterior oviduct and part of posterior uterus of adult female filled with sperm, left side view. (F) Adult female tail, ventral view, showing phasmids (ph) projecting through the thick cuticle. (G) Isolated spicules (sp) and gubernaculum (gub) of adult

male in ventral view. (H, I) Two different focal planes of the same male tail in ventral view, genital papillae labeled as in Fig 1H-J, showing a typical GP arrangement (i.e. ad+v4 close together and adcloacal). (J) Male tail in right side view showing variant with ad closer to v5 than to v4. Scale bars 10 µm for A–B, 50 µm for C–J.

Fig 3. Scanning electron micrographs of *Pellioditis pelhami* n. sp. strain EM434 female. (A) Habitus. (B–D) Lip region, *en face* view; note that the "bristle-like" features in C and D are bacteria. (E) Anterior region, left side view. (F) Lip region, ventral view. (G) Lateral field, vulval region with slit-like vulva, sublateral view. (H) Vulva, ventral view. (I) Lateral field, mid-body left-ventral view, vulva at left. (J–M) Posterior region showing conical tail, anus and slightly protruding phasmids, right side views (J, K), ventral view (L) and left side view (M). Abbreviations: dors. = dorsal, vent. = ventral, am = amphid, cs = cephalic sensillum, ils = inner lip sensillum, ols = outer lip sensillum, ph = phasmid.

Fig 4. Scanning electron micrographs of *Pellioditis pelhami* n. sp. strain EM434 adult male (A–K) and juvenile (sex unknown) (L–P). (A) Anterior portion, subventral view, showing lateral field; excretory pore (ep) at lower left and deirid (dei) at lower right. (B, C) Anterior end, *en face* view, showing lips separated into pairs: one dorsal and two sublateral. (D) Subventral view of anterior. (E–G) Lateral field. (H–K) Male tail, showing bursa, genital papillae, phasmids, spicules protruding from the cloaca, postcloacal sensilla. (H) Left sublateral view. (I) Ventral view. (J) Right lateral view. (K) Left lateral view. (L) *En face* view of juvenile mouth area, dorsal to the upper left. (M) Anterior of juvenile, left side view, dorsal to the right. (N) Lateral field of juvenile. (O, P) Juvenile tail, ventral and sublateral right side view, respectively, showing anus and slightly protruding phasmids. Scale bars 1 µm in B, C, L; 10 µm in other panels. Abbreviations: dors. = dorsal, vent. = ventral, am = amphid, cs = cephalic sensillum, ils = inner lip sensillum, ols = outer lip sensillum, ad = anterior dorsal genital papilla, pd = posterior dorsal genital papilla, ph = phasmid.

Measurements. See Table 1.

Table 1. Morphometrics of *Pellioditis pelhami* n. sp. strain EM434 and *Pellioditis pellio* strain SB361.

Character	*P. pelhami* sp. n. EM434 ♀[1,2]	*P. pellio* SB361 ♀	*P. pelhami* sp. n. EM434 ♂[1,2]	*P. pellio* SB361 ♂	*P. pelhami* sp. n. Holotype ♂	*P. pelhami* sp. n. Paratype♀	*P. pellio* Neotype ♂	*P. pellio* Topotype ♀
n	15	20	18	15				
L	1541 ± 122 (1302–1755)	1496 ± 112 (1302–1698)	1155 ± 70 (1041–1313)	1067 ± 57 (974–1155)	1109	1302	1149	1506

Character	*P. pelhami* sp. n. EM434 ♀[1,2]	*P. pellio* SB361 ♀	*P. pelhami* sp. n. EM434 ♂[1,2]	*P. pellio* SB361 ♂	*P. pelhami* sp. n. Holotype ♂	*P. pelhami* sp. n. Paratype♀	*P. pellio* Neotype ♂	*P. pellio* Topotype ♀
a	13.8 ± 1.1<br>(12.3–16.2)	18.7 ± 2.5<br>(15.0–25.0)	15.4 ± 1.3<br>(12.3–17.5)	20.4 ± 2.1<br>(17.2–24.3)	16.0	13.1	17.6	21.0
b	8.0 ± 0.04<br>(6.6–7.8)	7.59 ± 0.66<br>(6.7–9.0)	6.7 ± 0.6<br>(5.9–8.5)	6.0 ± 0.3<br>(5.7–6.4)	6.3	7.2	6.3	7.4
c	14.2 ± 1.2<br>(11.5–16.6)	13.1 ± 1.2<br>(10.8–15.2)	33.5 ± 3.3<br>(25.6–38.3)	31.4 ± 2.7<br>(26.6–34.8)	31	11.5	34.8	13.8
c'	3.1 ± 0.3<br>(2.7–3.6)	4.13 ± 0.5<br>(3.4–5.4)	1.0 ± 0.01<br>(0.9–1.4)	1.0 ± 0.08<br>(1.0–1.2)	1.2	3.4	1	3.9
V	51.9 ± 1.1<br>(48.2–52.9)	52.7 ± 0.2<br>(45.2–54.4)	–<br>–	–<br>–	–	51.7	–	54.1
Max. body diam.	112.7 ± 14.5<br>(94.3–142.4)	81.1 ± 9.5<br>(67.9–103.7)	75.4 ± 6.5<br>(64.1–90.5)	53.0 ± 7.9<br>(42.4–67.0)	73.6	99	65.1	71.7
Lip region diam.	12.1 ± 1.7<br>(8.5–15.1)	15.4 ± 1.2<br>(13.2–17.0)	12.3 ± 1.2<br>(11.3–15.1)	13.2 ± 0.7<br>(11.3–14.5)	11.3	12.3	14.1	15.1
Stoma L	17.7 ± 1.5<br>(14.1–19.8)	21.9 ± 1.6<br>(18.9–24.5)	16.5 ± 1.1<br>(14.1–17.9)	20.1 ± 0.7<br>(18.9–20.8)	18.0	18.9	20.7	22.6
Cheilostom L	5.5 ± 0.4<br>(4.7–5.7)	4.7 ± 0.8<br>(3.8–5.7)	5.2 ± 0.5<br>(4.7–5.7)	4.5 ± 0.7<br>(3.8–5.7)	5.7	5.7	4.7	3.8
Gymnostom L	4.0 ± 0.6<br>(2.8–4.7)	6.4 ± 0.9<br>(4.7–7.5)	3.4 ± 0.6<br>(2.8–4.7)	6.0 ± 0.9<br>(4.7–7.5)	3.8	3.8	5.7	4.7
Stegostom L	8.2 ± 1.3<br>(5.7–10.4)	10.8 ± 2.0<br>(7.5–14.1)	8.5± 2.5<br>(6.6–17.0)	9.6 ± 0.5<br>(8.5–10.4)	8.5	9.4	10.4	14.1
Procorpus L	55.9 ± 4.8<br>(49.0–66.0)	59.4 ± 3.9<br>(50.0–64.1)	49.2 ± 3.2<br>(40.5–53.8)	56.2 ± 3.7<br>(51.9–62.2)	48.1	49.0	55.6	61.2

Character	*P. pelhami* sp. n. EM434 ♀[1,2]	*P. pellio* SB361 ♀	*P. pelhami* sp. n. EM434 ♂[1,2]	*P. pellio* SB361 ♂	*P. pelhami* sp. n. Holotype ♂	*P. pelhami* sp. n. Paratype♀	*P. pellio* Neotype ♂	*P. pellio* Topotype ♀
Metacorpus L	29.4 ± 2.9	36.7 ± 3.7	26.8 ± 4.4	31.4 ± 2.1	28.3	28.3	33.0	33.0
	(26.4–35.8)	(29.2–43.4)	(21.7–41.5)	(27.4–34.0)				
Cardia L	15.2 ± 4.5	8.3 ± 3.1	16.3 ± 5.3	7.6 ± 1.6	20.7	15.1	10.3	4.7
	(7.5–21.7)	(4.7–17.9)	(6.6–24.5)	(4.7–10.4)				
Corpus L	85 ± 7	96 ± 5.4	76 ± 5.4	87 ± 3.8	76.4	77.3	88.6	94.3
	(77–101)	(85.8–104.7)	(66.0–88.6)	(79.2–95.2)				
Metacorpus diam.	23.6 ± 2.2	22.4 ± 2.2	20.9 ± 2.0	19 ± 1.3	18.9	21.7	17.9	22.6
	(20.7–28.3)	(19.8–27.3)	(17.0–24.5)	(17.9–22.6)				
Isthmus L	48.6 ± 5.1	38.9 ± 3.7	44.9 ± 5.1	35 ± 2.7	47.2	45.3	36.8	37.7
	(42.4–58.5)	(30.2–45.3)	(31.1–52.8)	(32.1–39.6)				
Basal bulb L	42.7 ± 2.8	40.7 ± 3.7	38.3 ± 2.6	33 ± 3.2	34	44.3	35.8	48.1
	(38.7–49.0)	(33.0–48.1)	(33.9–42.4)	(29.2–37.7)				
Basal bulb diam.	34.0± 2.8	30.4± 1.6	28.0± 2.3	26 ± 2.0	29.2	30.2	27.3	30.2
	(30.2–39.6)	(26.4–33.0)	(21.7–31.1)	(21.7–29.3)				
Pharynx L	193 ± 10.7	197 ± 7.4	173 ± 9.1	176 ± 6.4	176.3	182.0	182.0	202.8
	(179–213)	(188–213)	(153–190)	(164–185)				
Nerve ring position	131 ± 9.7	143 ± 8.5	116 ± 5.6	129 ± 4.5	121.7	126.4	128.2	148.1
	(116–154)	(129–159)	(102–126)	(119–137)				
E-pore position	172 ± 14.4	182 ± 7.2	167 ± 10.8	160 ± 8.5	162.2	160.3	174.4	190.5
	(159–192)	(171–196)	(149–186)	(148–174)				
Vagina L	60.4 ± 9.5	28.6 ± 9.0	–	–	–	42.4	–	34.9
	(23.3–33.6)	(12.3–44.3)						
G1 (% G)	52.8 ± 0.03	59.9 ± 0.1	–	–	–	47.8	–	60.8
	(47.8–56.0)	(50.0–76.3)						
G2 (% G)	47.2 ± 0.03	41.0 ± 0.04	–	–	–	52.2	–	39.2
	(44.0–52.2)	(35.0–50.0)						

Character	*P. pelhami* sp. n. EM434 ♀[1,2]	*P. pellio* SB361 ♀	*P. pelhami* sp. n. EM434 ♂[1,2]	*P. pellio* SB361 ♂	*P. pelhami* sp. n. Holotype ♂	*P. pelhami* sp. n. Paratype♀	*P. pellio* Neotype ♂	*P. pellio* Topotype ♀
Rectum L	34.4 ± 2.6 (28.3–37.7)	28.5 ± 4.1 (21.7–34.9)	–	–	–	33.0	–	30.2
Anal body diam. (ABD)	35.7 ± 3.9 (28.3–42.4)	28.0 ± 3.1 (20.7–31.1)	33.9 ± 2.6 (29.2–36.8)	32.7 ± 2.3 (28.3–35.8)	30.2	33.0	33.0	28.3
Anus to phasmid	40.1 ± 6.5 (31.1–50.9)	34.4± 4.9 (28.3–47.2)	–	–	–	38.7	–	32.1
Tail L	108.4 ± 8.6 (91.5–119.8)	114.2 ± 6.3 (103.7–125.4)	34.8 ± 3.1 (30.2–43.4)	34.2 ± 2.9 (30.2–39.6)	35.8	113.2	33.0	109.4
St L/LRW	1.5 ± 0.2 (1.2–2.0)	1.42 ± 0.1 (1.1–1.6)	1.4 ± 0.2 (1.1–1.6)	1.5 ± 0.1 (1.4–1.8)	1.6	1.5	1.5	1.5
Corpus L / isthmus L	1.77 ± 0.2 (1.5–2.4)	2.5 ± 0.3 (2.0–3.2)	1.7± 0.3 (1.5–2.8)	2.5± 0.2 (2.2–2.8)	1.6	1.6	2.4	2.6
Nerve ring (% PL)	68.3 ± 0.03 (61.1–72.6)	72.7 ± 0.03 (66.8–79.7)	67.2 ± 0.03 (61.9–74.6)	73.1 ± 0.02 (70.5–75.9)	69.0	64.1	70.5	73.0
E-pore (% PL)	90.6 ± 0.03 (87.8–97.1)	92.1 ± 0.02 (86.7–96.7)	95.1 ± 0.05 (85.0–100.0)	90.6 ± 0.04 (84.4–96.3)	92.0	69.4	95.8	94.0
G (no flex as % L)	57.6 ± 4.2 (52.4–65.3)	45.4 ± 0.7 (25.1–57.0)	–	–	–	58.3	–	45.1
Rectum L / ABD	1.0 ± 0.1 (0.8–1.3)	1.03 ± 0.2 (0.8–1.4)	–	–	–	1.0	–	1.1
Phasmid (% TL)	37.2 ± 6.7 (30.3–50.5)	24.5 ± 0.04 (24.2–39.4)	–	–	–	34.2	–	29.3
Spicule L	–	–	60.4 ± 5.1 (48.1–67.9)	58.5 ± 3.0 (53.8–62.2)	48.1	–	62.2	–

Character	*P. pelhami* sp. n. EM434 ♀[1,2]	*P. pellio* SB361 ♀	*P. pelhami* sp. n. EM434 ♂[1,2]	*P. pellio* SB361 ♂	*P. pelhami* sp. n. Holotype ♂	*P. pelhami* sp. n. Paratype♀	*P. pellio* Neotype ♂	*P. pellio* Topotype ♀
Gubernac-ulum L	–	–	27.7 ± 2.9 (21.7–31.1)	19.0 ± 2.4 (15.1–23.6)	21.7	–	19.8	–

[1] Grown on nematode growth medium with *Ochrobactrum* sp.

[2] Measurements were made from 4% formalin-fixed specimens processed to anhydrous glycerin, following a modified procedure of Seinhorst [25] as modified by De Grisse [24]. G_1/G_2: Vulva to anterior/posterior flexure of gonad as % of body length in female; corpus length (CL): measured along curvature of the lumen; neck length (NL): from anterior end to the base of the basal bulb, measured along middle of the body; reproductive tract length (RTL): measured along body axis, from anterior-most tip to posterior-most tip, *i.e*., excluding all flexures; stoma length: from cheilorhabdia to base of the stoma. Stoma terminology (cheilostom, gymnostom, and stegostom) was adapted from De Ley *et al.* [59], and terminology associated with the structures of the nematode anterior is based on Rashid *et al.* [60].

[3] After Hooper *et al*. [61].

[4] Equivalent to pharynx measurement in Hooper *et al.* [61]

Adults. Body cylindrical, almost straight to slightly curved ventrad in the middle when relaxed (Fig 3A). Cuticle about 4 µm thick. Annules fine in SEM images, inconspicuous under the light microscope. Lateral field with ca. 5-8 ridges (Figs 1G, 2A, 3G, 4E–G). Anterior end bluntly rounded, lip region continuous with body. Oral aperture triangular, with slightly convex sides and surrounded by six lips grouped in pairs (Figs 3B–F, 4B–C). Inner labial sensilla with protruding dendrite. Outer labial sensilla visible on SEM images, and sometimes in the light microscope as small "bumps" under the cuticle (Figs 3B, 4B). Cephalic sensilla present on dorsal and subventral lips (Figs 3B, 4B). Amphid opening a small inconspicuous slit on a small round mound (Figs 3B–D, 4B). Stoma short, only slightly longer than lip region is wide. Cheilostom indistinct, gymnostom and pro- and mesostegostom of about the same length (Figs 1D, 2B). Stegostom slightly broader at posterior, ending in well-developed rounded, isotopic metarhabdions, each with several minute tubercles, one central tubercle often more prominent (Figs 1D–E, 2B). Stoma is wider and shorter in adults than in juveniles (cf. Fig 1F), but with similar proportions between stegostom and gymnostom.

Pharynx corpus cylindrical, about two times as long as isthmus with slightly enlarged metacorpus narrowing into isthmus and a bulbous postcorpus (basal bulb) that is longer than wide, with striated valvular apparatus; isthmus slightly longer than basal bulb (Fig 1A, 2C). Nerve ring encircling isthmus at around 68% of pharynx length. Deirids conspicuous at same level or slightly anterior of excretory pore (Fig 4A). Excretory pore opening at mid-to-base of basal bulb (Fig 1A). Cardia conoid, highly variable.

Female. With the characters of adults. Reproductive system didelphic amphidelphic, ovaries reflexed with tips sometimes reaching the level of or past the vulva. Numerous sperm in a long oviduct that has a valve towards ovary and uterus, respectively, forming a spermatheca (Fig 2E). Sperm is also found in the uteri. Sperm diameter ca. 10 µm. Uteri of mature females are often filled with developing embryos. In older females, hatched juveniles of various stages are also present inside of the female uterus. Eggs oval, measuring ca. 50x30 µm. Vulva a transverse slit halfway (50–54%) along the body (Figs 3A, G–I). Vagina length variable, often extending past middle of vulval body diameter. Intestine ending in a rectum of about equal length as anal body width (ABW, Fig 1B). Three rectal gland cells visible. Anus an arcuate slit (Figs 3J–M). Phasmids prominent, forming small protruding papillae, located at 40 (31–51)% of tail length. Tail conical, with acute tip. (Figs 1B–C, 2F, 3J–M).

Male. Body habitus similar to female when relaxed, but shorter and narrower. Reproductive system monorchic, occupying 60-70% of total body length; testis ventrally reflexed. Spicules separate with furcated tip, spicule head not set off (Figs 1H–I, K, 2G, 4H–K). Gubernaculum spatula-shaped in dorso-ventral view (Figs 1L, 2G). Bursa relatively narrow, anteriorly open, anterior margin slightly wavy (Figs 1H–I, 2H–J, 4H–K). Nine pairs of genital papillae (GPs, “rays”). Three precloacal GPs. The tip of the first GP (v1) opens at the edge of the fan. The anterior dorsal GP is in position 5 (counting from the anterior). The posterior dorsal GP is terminal, or it originates at the same level as the most posterior GP (v7). The posterior three GPs often form a group. The phasmids form small ventral papillae that are not fully embedded in the bursal velum. Thus, the formula (following [11, 29]), is: v1,v2,v3/(v4,ad)/v5,(v6,v7,pd),ph or v1,v2,v3/(v4,ad)/v5,v6,(v7,pd),ph (where 'ad' and 'pd' represent the anterior and posterior dorsal papillae homologous across rhabditids [23, 27], 'v' represents the other papillae, 'ph' represents the phasmid; here,

two slashes '/' represent the adcloacal position of v4+ad). This pattern is also revealed via immunofluorescent staining of the neuroepithelial adherens junctions in J4 juveniles (Fig 1J) at the time when papillae form in the external cuticle (Fig 2D). In strain EM434, variations in the spacing of GP1, GP2 and GP3 are observed. Aberrations are also found: males with abnormal numbers of rays (one having 13 GPs on one side!) and one adult male with a long (leptoderan) tail tip. Cloacal aperture arcuate slit-shaped, its anterior edge slightly protruding, precloacal sensillum inconspicuous, postcloacal sensilla fairly long with a broad base (Figs 1H, 4H–K). Tail peloderan.

Type locality and habitat. Strain EM434 was started by DHAF in 1990 from a single gravid female from an unidentified earthworm collected by S. Baird on the sidewalk in front of the Forschheimer Building of Albert Einstein College of Medicine (1300 Morris Park Ave., Bronx, NY, USA; GPS coordinates 40.850969, -73.845843). DF5056 was similarly obtained by DHAF in 1993 from an earthworm in lawn soil on City Island (515 Minnieford Ave., Bronx, NY, USA; GPS coordinates 40.8525633, -73.7889098). Neither earthworms were identified at the time, but were similar in appearance to *Lumbricus rubellus*. In conformity with the system used by the *Caenorhabditis* Genetics Center (CGC), these strains were given the strain designations EM434 and DF5056.

Type material and living cultures. Of strain EM434, the male holotype (measurements in Table 1) plus another male and two female paratypes (not measured) have been deposited at the Wageningen Nematode Collection, National Plant Protection Organization, Wageningen, The Netherlands, as slide WT3938. The slide contains two females and two males; the rightmost male is the holotype. Another slide containing the paratype represented in Table 1 has been deposited with the Nematode Collection at the University of California, Davis as slide ____. This slide contains six female paratypes; the fourth (counting left-to-right) is the measured paratype. A third slide with two female and two male paratypes has been deposited as slide UGMD_104442 at the Nematode Collection, Ghent University Zoology Museum. Measurements were obtained for the rightmost male (data not shown). Live cultures of EM434 are cryogenically archived at the *Caenorhabditis* Genetics Center (CGC), University of Minnesota, and at the New York University Rhabditid Collection (NYURC).

DNA sequences deposited at GenBank for strain EM434:

OR059186 (newly reported here): nearly complete repeat unit for the nuclear ribosomal RNA genes (18S, ITS1, 5.8S, ITS2, 28S)

EU161967: RNA polymerase 2 gene, largest subunit, partial CDS

EU196008: 18S ribosomal RNA gene, partial sequence

EU195967: 28S ribosomal RNA gene, partial sequence

MT472231: mitochondrial 16S ribosomal RNA gene, partial sequence, and NADH dehydrogenase subunit 3 (ND3) gene, partial CDS

MT472187: mitochondrial tRNA-Glu gene and 12S ribosomal RNA gene, partial sequence

Pellioditis pellio (Schneider, 1866)

ZooBank LSID: urn:lsid:zoobank.org:act:C1CDBEEF-A1BB-4B0A-AF6A-BC8051416FD8

Species names synonyms:

= *Phasmarhabditis bohemica* Nermuť, Půža, Mekete & Mráček, 2017 syn. nov.

= *Pellioditis bohemica* (Nermuť, Půža, Mekete & Mráček, 2017) Sudhaus, 2023 syn. nov.

Strains:

- SB361: leg. W. Sudhaus, from a *Lumbricus rubellus* Hoffmeister, 1843, found in dry ground in a ruderal area (52.41500, 13.19870) in March, 2004, in Berlin, Germany, corresponding to the type location for this species [18]. The strain was cultured on water agar plates supplemented with small pieces of beef; first cryogenically preserved June 17, 2005, by K. Kiontke.
- CH1: leg. J. Nermut' in summer, 2012, from a gray slug, *Deroceras reticulatum* Müller, 1774, from a redcurrant plantation near the village of Chelčice, Czech Republic (49.1287125, 14.1739622), altitude 450 m above sea level [6]. SSU rDNA sequences of haplotype 1 of CH1 and SB361 are identical (haplotype 2 is different from haplotype 1 by 11 nucleotides in a cluster); LSU rDNA and

ITS sequences of CH1 and SB361 are essentially identical, except for 2 ambiguities and 1 SNP.

- 185: leg. J. Nermut' in summer, 2012, from a gray slug, *Deroceras reticulatum* Müller, 1774, from a redcurrant plantation near the village of Chelčice, Czech Republic, close to where CH1 was isolated [6]. LSU rDNA and ITS sequences of CH1, 185 and SB361 are essentially identical, except for 2 ambiguities and 1 SNP.

Description

Based on strain SB361.

Illustrations. Figs 5–8, supplemental videos showing stacks of DIC focal planes through live animals (S10 Video–S19 Video).

Fig 5. Drawings of *Pellioditis pellio* strain SB361 female (A-E) and male (F-H). (A) Anterior portion showing stoma, pharynx and excretory pore, left side view. (B, C) Female tail showing positions of anus and phasmids; left side (B) and ventral (C) views. (D) Stoma region, right side view. (E) Lateral field near the midbody. (F) Ventral view of adult male tail with genital papillae labeled according to the rhabditid homology system [28, 29] based on common developmental origins of the GPs in the lateral epidermis [23, 27, 57, 58]. (G) Spicule (lateral view) and (H) gubernaculum (ventral view). Scale bars as shown.

Fig 6. DIC photomicrographs of *P. pellio*. (A) Anterior uterus and oviduct of a young female and two laid embryos, arrow points to the position of the vulva. (B) Female lateral field. (C) Female tail in right side view. (D) Pharynx region of an adult male, right side view; arrow points to the location of the excretory pore. (E) Stoma of an adult female in right side view; arrow points to the border between gymnostom and stegostom. (F) Isolated spicules in lateral view and gubernaculum in ventral view. (G) Male tail in right side view. Anterior (ad) and posterior dorsal (pd) genital papillae are indicated with arrows. (H) Male tail in ventral view; the base of the postcloacal sensilla is in focus here (unlabeled arrows). (I) Male tail in ventral view, focused closer to the body than in H and showing phasmids (ph) and the distal end of the gubernaculum (gub). Scale bars 10 µm in B and E, all others 50 µm.

Fig 7. SEM micrographs of *Pellioditis pellio* females (A, B, E, J, K, O, P) and males (C, D, F-I, L-N). (A, B) Lip region

of female, *en face* view; in A, the right dorsal lip is abnormal. (C, D) Lip region of male, *en face* view. (E) Anterior portion of a female in subventral right view. (F) anterior portion of a male in subventral left view, (G–I) enlarged detail views corresponding to areas delineated in F. (J) Vulval region of a female with mating plug; inset shows detail of lateral field. (K) Female vulva. (L–N) Male tail in ventral or subventral view showing narrow bursa with genital papillae and cloacal opening with postcloacal sensillum. (O, P) Female tail in ventral and left side views. The phasmids (ph) form small papillae. Abbreviations: am = amphid opening, cs = cephalic sensilla, ep = excretory pore, ad = anterior dorsal genital papilla, pd = posterior dorsal genital papilla, ph = phasmid. Scale bars 10 µM.

Fig 8. DIC photomicrographs comparing buccal tube and spicules of *Pellioditis pelhami* n. sp. and *Pellioditis pellio*. *P. pelhami* n. sp. and *P. pellio* differ in the shape of the stoma and in the shape of the spicule tip. The stoma of *P. pelhami* n. sp. is shorter and wider—and the spicule tip is narrower—than those of *P. pellio*. (A–D) *P. pelhami* n. sp. (E–H) *P. pellio*. (A) Stoma of an adult female in subventral left view. (B) Stoma of an adult male in subventral left view. (E) Stoma of an adult female right side view. (F) Stoma of an adult male right side view. (C, D, G, H) Spicules in lateral view; gubernaculum in ventral view. Scale bars for all stoma images 10 µm (A, B, E, F), for all spicule images 20 µm (C, D, G, H).

Measurements. See Table 1.

Adult females and males. Females and males are very similar to *P. pelhami* n. sp., but about 5% longer and 40% wider at maximum body width when grown under the same conditions. A clear difference is found in the stoma, which is consistently longer and narrower in *P. pellio*: the stoma of *P. pellio* is about 4.5 times as long as wide (Figs 5D, 6E), whereas that of *P. pelhami* n. sp. is less than 3 times as long as wide (Figs 1D, 2B, 8A–B). A subtle difference is seen in the tip of the spicule, which is narrower in *P. pelhami* n. sp. than in *P. pellio* (Figs 8C-D, G-H). In strain SB361, there is variation in ray positions such that GP v4 and the anterior dorsal GP may form a close group (Figs 5F, 6G–I, 7L–N) or not (Fig 6H). Aberrations with fused or missing GPs are frequently observed. All other dimensions and morphological features are essentially identical in both species.

Life cycle. Here, we synthesize several observations of the *P. pellio* life cycle. *P. pellio* is a necromenic bacteriophagic nematode, whose dauer juveniles invade nephridia, seminal vesicles or the

coelom of different earthworm species or become encapsulated in brown bodies [9, 62]. There, they wait for the earthworm to die and develop on the cadaver or develop in the decaying terminal end of the worm when it is detached by autotomy [63]. Dauer juveniles do not wave and do not survive dessication. However, they have survived in water 4½ months at room temperature [64] and 24 months at 2.5 ˚C (WS, unpub. observations). Males are nearly as frequent as females (43–48%). Copulation follows the parallel type: a mating plug is deposited (Fig 7J). The species is ovoviviparous or juveniles develop inside the mother up to the dauer stage. Development takes 3.5–4 days at 20 ˚C, generation time is 4–5 days, life span of adults 5–10 days. Each female can have hundreds of offspring; e.g. 570 offspring were reported from one female mated several times [65].

Identification. Strain SB361 is no different from the original description of *P. pellio* [18]. Like the original isolate, it was obtained from a gray earthworm from the type location (Berlin, Germany); morphological features are identical: e.g. a conical female tail with a thick cuticle and prominent phasmids, male ray and bursa morphologies. *P. pellio* is reported to be commonly isolated from earthworms [9, 18, 66-68] and is likely necromenic on them [11]. Thus, strain SB361—isolated from the type location and host—represents *P. pellio*.

Fig 9. Comparison of male and female tails of *Pellioditis pellio* redescribed here with those depicted in figures from the original description (Schneider, 1866) [18], subsequent plausible reisolates and isolates mistakenly claimed to be *P. pellio*. Schneider [18] only provided a single drawing in the original description: a female tail in ventral view. In the description of *P. pellio*, he noted that the species was in all aspects similar to his directly preceding description of *P. papillosa* (of which the male bursa is redrawn here from Taf. XI, Fig IIIc [18]), except that the female tail of *P. papillosa* was cupola-shaped whereas that of *P. pellio* was cone-shaped (redrawn here from Taf. XI, Fig X [18]). Neither Schneider nor any of the other authors depicted the phasmids in the males, which are admittedly not very prominent in this species and could have easily been overlooked. In all of the depictions of species compatible with the original description (e.g., from the current paper, Reiter [67], Völk [68], Örley [66], and Hertwig [9]), the male tail is peloderan and GPs 4-5 (i.e., v4 and ad) are situated close together, at about the same anterioposterior location as the cloaca. Also, wherever female tails are depicted in these descriptions, all show conical forms, though of varying lengths consistent with variation present in strain SB361 (Table 1). In contrast, the male tails from other depictions mistakenly identified as *P. pellio* (bottom row)

have clearly different male tail tip morphologies and papilla arrangements. For example, the drawings by Bütschli [69] and Thorne [70] show species with leptoderan tail tips; those by Bütschli [69] and Aubertot [71] show GPs 2-3 bracketing the cloaca and those by Thorne [70] and Stock & Camino [72] show GPs 3-4 bracketing the cloaca.

Voucher material. Of strain SB361, a slide with the neotype male (morphometrics in Table 1) has been deposited at Wageningen Nematode Collection as slide WT3937. This slide has three males and three females from the series; the neotype is the leftmost male. A slide with the measured topotype female (Table 1) has been deposited with Ghent University Zoology Museum, as slide UGMD_104443. This slide has three females and three males from the series; the measured topotype female is leftmost. A third slide with 3 males and four females of this series has been deposited as slide ____ at the Nematode Collection, University of California, Davis. The second female from the left has been measured (data not shown). Live cultures of SB361 are cryogenically archived at the CGC and the NYURC.

DNA sequences deposited at GenBank for SB361. OR059187 (newly reported here): nearly complete repeat unit for the nuclear ribosomal RNA genes (18S, ITS1, 5.8S, ITS2, 28S)

Differential diagnosis

P. pelhami n. sp. and *P. pellio* can be distinguished from each other and from all known species of the *Pellioditis* clade. As noted above, females and males of *P. pellio* are slightly larger than *P. pelhami* n. sp. when grown under the same conditions. The stoma is shorter and wider in *P. pelhami* n. sp. than in *P. pellio* (Figs 8A–B vs. 8E–F) and the spicule tip is slightly narrower in *P. pelhami* n. sp. than in *P. pellio* (Figs 8C–D vs. 8G–H). We also performed reciprocal crosses and found them to be reproductively isolated from each other (Table 2).

Table 2. Interfertility tests.[1]

	No males	EM434 males	SB361 males

EM434 females	many oocytes laid without eggshells	many juveniles (and some young adults)	eggs only (with eggshells)
SB361 females	no oocytes detected	eggs with eggshells and some oocytes	many juveniles (and some young adults)

[1] Three or four females from one strain were left uncrossed (control to test their virginity), crossed with three or four males from the same strain (positive control), or crossed with three or four males from the other strain (duplicates were set for these latter crosses). Late J4 or early adult individuals were used to set the crosses. Plates were kept at 20 ˚C and scored 4 days later. These crosses demonstrate: (1) *Pellioditis pelhami* EM434 is reproductively isolated from *Pellioditis pellio* SB361, and (2) isolation is possibly postzygotic, as sperm from each species were able to induce egg maturation. We did not check for embryonic development, however.

Two species of *Pellioditis* have only rare or no males: *P. hermaphrodita* and *P. californica* [53], whereas approximately half of *P. pelhami* n. sp. and *P. pellio* populations are males (although a parthenogenetic variant of *P. pellio* has been documented [9]).

In contrast to the conical-shaped female tails of *P. pelhami* n. sp. and *P. pellio*, many *Pellioditis* species have female tails that are moderately or strongly cupola-shaped (dome-shaped with a pointed spike projecting from the "top" of the dome): *P. akhaldaba*, *P. bonaquensis*, *P. huizhouensis*, *P. meridionalis*, *P. papillosa*, *P. safricana*, and *P. zhejiangensis*. From its description [73], it appears that *P. akhaldaba* actually encompasses two different but closely related species (see the list of valid species below). One form ("slow") has mostly cupola-shaped tails, whereas the other form ("quick") has conoid-tails. This latter form can be distinguished from *P. pelhami* n. sp. and *P. pellio* by having, for example, different DNA sequences and a lateral field with three strong ridges (instead of ≥5).

P. pellio and *P. pelhami* n. sp. can also be differentiated from other gonochoristic species having conical or conoid female tails, i.e. *P. apuliae*, *P. bohemica*, *P. circassica*, *P. clausiliiae*, *P. kenyaensis*, *P. neopapillosa*, *P. quinamensis*, *P. thesamica*, and *P. tawfiki*. *P. tawfiki* males have GP1 and GP2 very close together, and only these GPs are precloacal [74]; Sudhaus [75] has previously noted this species fits better in *Pelodera* instead of *Pellioditis*. In males of *P. kenyaensis* [47], GP4 and GP5 are the same distance apart

as GP3 and GP4, different from the usually close apposition between GP4 and GP5 in *P. pelhami* n. sp. and *P. pellio*. In *P. thesamica* [55], GP1 and GP2 are considerably further apart than are GP2 and GP3, whereas these distances are similar in most individuals of *P. pelhami* n. sp. and *P. pellio*. In *P. quinamensis* [46], each of the three pairs of lips are strongly fused and separated from the other pairs, different from our strains where there is distinct separation between lips of each pair. Four species, *P. apuliae*, *P. bohemica*, *P. circassica*, and *P. clausiliiae* [6, 16, 45], all have a similar GP formula and can only be distinguished by DNA sequences and morphometrics. Two of these, *P. circassica* and *P. clausiliiae* have the shortest female tails and the shortest male spicules (<41 µm) [45]; all the others, *P. apuliae*, *P. bohemica*, *P. pelhami* n. sp. and *P. pellio*, have longer female tails on average and much longer male spicules (>48 µm). *P. apuliae* [16] and *P. pelhami* n. sp. have short, wide stomas (length/width ratios <3), whereas *P. bohemica*, *P. pellio* and the others have longer stomas (length/width ratios >3).

Thus, the only two species not differentiable on morphological grounds are *P. bohemica* and *P. pellio*. As detailed below, they are also not differentiable at the molecular level and are therefore synonymous. That is, *P. bohemica* is a junior synonym of *P. pellio*. The fact that *P. bohemica* was collected from slugs accords with previous accounts of *P. pellio* being isolated from slugs as well as earthworms [5, 10, 64].

One further species to compare, *Pellioditis mairei* [5], is poorly described, with only a figure of the male tail and measurements for one female. Drawings of the male bursa look essentially identical to those of *P. pellio*. Two characters that distinguish *P. mairei* from *P. pellio* (and strain SB361) as well as *P. pelhami* are the much larger size of *P. mairei* females (3400 µm vs. ca. 1500 µm) and the relatively shorter (presumably conoid) female tail (c = 23.6 for *P. mairei* vs. ca. 13–14). Strain DD of *P. mairei* was also isolated from an earthworm, but from a garden in Algiers [5]. Reciprocal crossing experiments showed that *P. mairei* was postzygotically isolated from *P. pellio* collected from slugs [2, 5].

Notes on erroneous and plausible records of *P. pellio* in the literature

Because we re-isolated *P. pellio* from its type location and could study its characters carefully, we can scrutinize previous records of *P. pellio* (Fig 9). For early nematologists, a preferred method for obtaining rhabditid nematodes was to chop earthworms into heat-treated soil and to wait for necromenic nematodes to develop on the putrefying cadaver. Often, these authors assumed that the nematodes they isolated were *P. pellio* without checking morphological characters, even though several species of rhabditids are associated with earthworms and *P. pellio* is not even the most frequent one [76]. Fortunately, some authors provided figures or references to characters, thus allowing subsequent identification of the actual species under investigation. For example, many authors who referred to a species called *"Rhabditis pellio"* as described by Bütschli, 1873 [69] actually must have been working with *Rhabditis maupasi* Seurat in Maupas, 1919 [5]. This species is clearly distinguished from *P. pellio* by a leptoderan versus peloderan male tail tip (Fig 9). The species investigated by Aubertot [71] under the name *"R. pellio"* can be identified via his clear illustration as *Reiterina typica* Stefański, 1922 (Fig 9). Consequently, all notes about *"R. pellio"* in the literature referring to Aubertot's observations that *"R. pellio*" has waving dauer juveniles and phoresis on *Drosophila* should instead be applied to *R. typica*. Another example is the "*Rhabditis (Pelloides) pellio*" (*Pelloides lapsus*) described by Thorne, 1961 (1961: 451; Fig 9) [70]. This specimen is clearly not *P. pellio* as originally described by Schneider, since it has a leptoderan male tail tip. Instead, it belongs to the *Maupasi* species group of *Rhabditis*. Because Thorne obtained his specimen from Dougherty [70], we also have to be somewhat skeptical that Dougherty & Calhoun [77] were working with *P. pellio*, even though Chitwood identified their culture as *"R. pellio"* (see footnote 2, p. 55, of Dougherty & Calhoun [77]). As a final example of misidentification, the alleged "*Pellioditis pellio*" isolated from tabanid larvae by Stock & Camino [72, 78] is in all likelihood an *Oscheius* species (see Fig 9).

On the other hand, several other investigators could have plausibly worked with the "true" *P. pellio*, or at least a very similar *Pellioditis* species. The illustrations associated with the studies of Reiter, Völk, Örley, and Hertwig [9, 66-68] all suggest they could well have been working with *P. pellio* (Fig 9). Even without illustrations, it is sometimes clear that *P. pellio* or a very closely related species was used. Poinar & Thomas [79] and Somers et al. [65] investigated a rhabditid they called *"Rhabditis pellio"* from the

earthworm *Aporrectodea trapezoides* in California. From their introduction, it is clear that these authors were aware of the differences between Bütschli's leptoderan "*R. pellio*" and the actual peloderan *P. pellio* of Schneider [79]. Poinar identified the species that Eveland et al. [80] used in their scanning electron microscopy investigation, which was thus likely *P. pellio* or a similar species. According to the male bursa and other characters they described, Goodchild & Irwin [81] plausibly identified an isolate similar to *P. pellio* from *Lumbricus* in Georgia and Illinois. However, their clear picture of the stoma shows a length/width ratio <3, more consistent with *P. pelhami* n. sp. than with *P. pellio* (length/width ratio 4.5). Similarly, Zaborski et al. [82] grew a nematode species from *Lumbricus terrestris* from Illinois that is similar to *P. pellio* in some respects, but has a short and wide stoma and is as large as *P. mairei* (female length >3 mm) [5].

Valid species in genus *Pellioditis* Dougherty, 1953

Pellioditis Dougherty, 1953: type species *Pelodera pellio* Schneider, 1866 [18, 83].

syn. *Phasmarhabditis* Andrássy, 1976: type species *Pelodera papillosa* Schneider, 1866 [11, 84]. Sudhaus [2, 11] previously combined *Pellioditis* instead of *Phasmarhabditis* with species names below.

- *P. akhaldaba* (Ivanova, Gorgadze, Lortkhipanidze & Spiridonov, 2021)—Akhaldaba, Georgia [73]. Note: It is likely that this species name was applied to two very similar but different species from the same locality, one from the slug *Deroceras reticulatum* and one from garden soil baited with *Galleria* (wax moth) larvae. The labeling of the different isolates as "quick" (from slugs) and "slow" (from soil) reflects a behavioral difference. The "quick" females have a conical tail whereas the tail is cupola-shaped in 80% of the "slow" females. The "quick" isolate is generally smaller than the "slow" one, and several measurements show significant differences between the isolates: stoma length, diameter of corpus and terminal bulb of the pharynx, flexure of the testis, distance between anus and phasmids in females and distance between lips and excretory pore in exsheathed dauer larvae.

Unfortunately, no crossing experiments were reported to test interisolate reproductive compatibility.

- *P. apuliae* (Nermuť, Půža & Mráček, 2016)—Bari, southern Italy; from the slug *Milax sowerbyi* in the garden of the University [16].
- *P. bonaquensis* (Nermuť, Půža, Mekete & Mráček, 2016) emendation of *Phasmarhabditis bonaquaense* Nermuť, Půža, Mekete & Mráček, 2016 by D. J. Hunt (footnote on p. 232 of [46])—České Švýcary, near Dobrá Voda, Czech Republic; from the slug *Malacolimax tenellus* [15].
- *P. californica* (Tandingan De Ley, Holovachov, Mc Donnell, Bert, Paine & De Ley, 2016)—Eureka, California, USA; obtained from a cadaver of the slug *Deroceras reticulatum* [53].
- *P. circassica* (Ivanova, Geraskina & Spiridonov, 2020)—Nickel settlement, Adygea Republic, Russia; from the snail *Oxychilus* cf. *difficilis*, in deciduous forest [45].
- *P. clausiliiae* (Ivanova, Geraskina & Spiridonov, 2020) —near Georgievsk, Stavropol district, Russia; from Clausiliidae snails (cf. *Quadriplicata* sp.) in decidous forest [45].
- *P. hermaphrodita* (Schneider, 1859)—Berlin, Germany; in a slug (or snail? “Schnecke”) [85].

  syn. *Leptodera foecunda* Schneider, 1866 [18]

  Rhabditis caussaneli Maupas, 1899—Vire, Normandy, France; dauer juveniles in the slug *Arion empiricorum* var. *ater* [64].
- *P. huizhouensis* (Huang, Ye, Ren & Zhao, 2015)—Kowloon Peak of Huizhou City, Guangdong Province, China; in rotting leaves [13].
- *P. incilaria* (Yokoo & Shinohara in Shinohara & Yokoo, 1958)—Kurume-Shi, Kyushu, Japan; isolated from the slug *Incilaria confusa* [86].

  syn. *Rhabditis fruticicolae* Kreis, 1967, *nec* Shinohara, 1960 [87]—Nagasaki, Japan; from the snail *Fruticicola sieboldiana* [88].
- *P. kenyaensis* (Pieterse, Rowson, Tiedt, Malan, Haukeland & Ross, 2021) —Nairobi, Kenya; from the slug *Polytoxon robustum* in a garden [47].
- *P. mairei* (Maupas, 1919)—locality not given, but likely in Algeria; dauer juveniles in a gray

earthworm from a garden [5].

- *P. meridionalis* (Ivanova & Spiridonov, 2017)—Cát Tiên National Park, southern Vietnam; from the land snail *Quantula striata* [89].
- *P. neopapillosa* (Mengert in Osche, 1952)—Bavaria (near Berchtesgaden or near Hersbruck), Germany; dauer juveniles in the keelback slug *Limax cinereoniger* [90].
- *P. papillosa* (Schneider, 1866)—Berlin, Germany; in decaying substance, dauer juveniles associated with the slug *Limax ater*; necromenic in slugs, Arionidae and Limacidae [18].

  syn. *Angiostoma limacis apud* Will (1848) [91], *nec* Dujardin, 1844 [92].

  Rhabditis ikedai Tadano, 1950—Sendai or Tokyo, Japan; from the slug *Incilaria confusa* [93].

  Rhabditis ninomiyai Yokoo, 1968—Nagasaki, Japan; from the alimentary organs of a snail (*Fruticicola* or *Euhadra*) [94].
- *P. pellio* (Schneider, 1866)—Berlin, Germany; dauer juveniles in the body cavity of a gray earthworm [18]. Due to carelessness, very often confused with other species from earthworms, especially *Rhabditis maupasi*. (See text above for prior mistaken identifications of *P. pellio*.)

  syn. nov. *Pellioditis bohemica* (Nermuť, Půža, Mekete & Mráček, 2017)—near Chelčice, Czech Republic; from *Deroceras reticulatum* from a red currant plantation; also from soil samples baited with *Galleria* larvae [6].
- *P. quinamensis* (Ivanova & Spiridonov, 2022)—Cát Tiên National Park, southern Vietnam; from the land snail *Sesara* sp. Further isolates from seven additional genus taxa of terrestrial snails in that National Park [46].
- *P. safricana* (Ross, Pieterse, Malan & Ivanova, 2018)—near George, Western Cape province, South Africa; from the slug *Deroceras reticulatum* from a nursery [52].
- *P. tawfiki* (Azzam, 2003)—Great Cairo, Egypt; unclear whether the type was isolated from the snail *Eobania vermiculata* or the slug *Limax flavus* [74]. Note: due to several described characters,

Sudhaus [2] suggests that this dubious species may instead belong in genus *Pelodera* (*Teres* species group).

- *P. thesamica* (Gorgadze, Troccoli, Fanelli, Tarasco & De Luca, 2022)—Tezami, East Georgia; from a dead *Deroceras reticulatum* slug in a raspberry garden [55].
- *P. villasmundi* (Ivanova, Clausi, Leone & Spiridonov. 2023)—Nature Reserve 'Speleological Complex Villasmundo—S. Alfio', Syracuse Province, Sicily; from the slug *Milax nigricans*, found in 7 additional gastropod species [95].
- *P. zhejiangensis* (Zhang & Liu, 2020)—Ningbo, Zhejiang province, China; from the slug *Meghimatium (= Philomycus) bilineatum* from a vegetable garden [96].

Phylogenetic relationships using DNA sequences

Most *Pellioditis* species (19 described and 5 undescribed) are represented by DNA sequences covering various segments of nuclear and mitochondrial ribosomal RNA genes, part of the gene for the large subunit of RNA polymerase II (minus introns), and/or the mitochondrial cytochrome oxidase I gene and other various mitochondrial genes. The phylogenetic diagrams that have been published with species descriptions since 2015 are usually only based on small sequence segments from individual genes and vary widely in the relationships depicted. While these analyses may serve to show genus-level membership of a new species, building a robust phylogenetic framework for testing evolutionary hypotheses requires much more extensive molecular data.

Here, we collected as many representative sequences as possible from GenBank (200; S1 Table) and constructed a DNA alignment supermatrix (see Materials and Methods for details). To this matrix, we added our two new nearly complete rDNA sequences for *P. pelhami* n. sp. EM434 and *P. pellio* SB361. We included an *Agfa* and an *Angiostoma* species, proposed to be closely related to *Pellioditis* [11, 28, 97], as well as representatives from nearly every genus in Rhabditidae for an outgroup sample. To test how robust the phylogenetic inference would be to methodology, we analyzed the data via two different approaches: (1)

weighted parsimony (WP) using the full dataset (all taxa with all characters), which contained a lot of gaps or missing data, and (2) maximum likelihood (ML) using taxon- and character-subsets of the supermatrix, each with few gaps, and then puzzling the resulting trees into a supertree. Bootstrapping (for ML) and jackknifing (for WP) were used to test how robust inferred bipartitions (phylogenetic branches) are to data sampling.

Although the ML approach provided less resolution than WP, phylogenies using both approaches are compatible in most respects (Fig 10A). Conflict is only apparent in how the three *Pellioditis* species groups are related (see below) and in two of the deeper relationships within the outgroup. These cladograms allow us to make the following observations:

Fig 10. Results of phylogenetic analyses (A) and evolutionary reconstructions (B). (A) Molecular phylogenetic analyses. Left tree: WP analysis of all parsimony-informative sites in the supermatrix, which consists of sequences from the nuclear ribosomal RNA gene cluster, the RNA polymerase large subunit gene, and mitochondrial sequences spanning the cytochrome oxidase I gene and the mitochondrial ribosomal RNA (and intervening) gene sequences (see S1 Fig). Numbers at nodes are percentages of 1000 jackknife replicates (25% of characters randomly deleted per replicate) that supported those bipartitions. Synrhabditis and *Pellioditis* clades [11] are strongly supported. Right tree: Clann [36] supertree of bootstrapped ML trees from 7 subsets of the supermatrix (see S1 Fig). Numbers at the nodes represent the proportions of bipartitions among individual ML trees were consistent with the branches shown. In both trees, branches are collapsed where bipartition support is less than 70% or 0.7, such that only robustly supported branches are shown. Four species groups are shown with different colors: *Pellio* group, *Papillosa* group, *Kenyaensis* group and a group containing *P. huizhouensis* and *P. meridionalis*. Obligate parasitism evolved twice, once in the Angiostomatidae (represented here by *Angiostoma norvegicus*) from within *Pellioditis*, and in the lineage to *Agfa* (represented here by *Agfa flexilis*), the sister group to *Pellioditis*. (B) Character evolution traced by parsimony onto the WP phylogeny. Cupola-shaped female tails have evolved four times independently from conical-shaped tails (red lineages), and possibly again in the "slow" isolate of *P. akhaldaba*, not shown. Hermaphroditism evolved twice: in the lineages to *P. hermaphrodita* and *P. californica*.

- *Agfa* is the sister taxon of a robustly supported clade that includes *Pellioditis* + Angiostomatidae (represented by *Angiostoma norvegicus*, but other *Angiostoma* sequences are very similar). Based

on the better-resolved WP phylogeny, Angiostomatidae likely branches off within *Pellioditis*. If this holds true, *Pellioditis* is paraphyletic with respect to this group of obligate parasites. The *Pellioditis* sister taxon of Angiostomatidae, however, is at present unresolved.

- There are three robustly supported clades within *Pellioditis*: a small group that contains only *P. huizhouensis* and *P. meridionalis*, a group of mostly South African species (*Kenyaensis* species group), and a group that includes *P. papillosa*, *P. californica* and *P. pelhami* n. sp., among others (*Papillosa* species group). A fourth group that is well supported by WP but not by ML includes *P. pellio*, *P. hermaphrodita*, and *P. neopapillosa*, among others (tentatively called the *Pellio* group). Angiostomatidae may be sister to or derived from within this latter group.
- *P. hermaphrodita* and *P. neopapillosa* are likely sister species. It is interesting to note this species pair was suggested early on by Osche [98] as an example of "complementary" species [28]. In this scenario of sympatric speciation from a gonochoristic stemspecies, a uniparental (parthenogenetic or self-hermaphroditic) species splits from and thus has a gonochoristic sister species, with which it co-exists. A parthenogenetic variant was reported from an otherwise gonochoristic *P. pellio* population [9]. Also, the fact that *P. hermaprhodita* and *P. californica* are derived from separate clades shows that their hermaphroditic mode of reproduction arose independently (Fig 10B). Hermaphroditism and other modes of uniparental reproduction have arisen multiple times in rhabditids, possibly facilitated by the architecture of the sex determination pathway as well as gene duplications unique to particular lineages [20, 21, 99].
- For both trees depicted in Fig 10A, a conoid female tail is plesiomorphic for *Pellioditis* (Fig 10B). Parsimony reconstruction suggests that cupola-shaped tails originated at least four times. Thus, this morphological feature appears to be quite homoplasious in this group.
- *P. pellio* and *P. pelhami* n. sp. are in separate clades, suggesting that associations with earthworms arose multiple times, not just once as proposed previously [75]. An alternative interpretation, however, is that associations with both slugs and earthworms are plesiomorphic but that there is an

ascertainment bias in reporting the host: i.e., investigations of only slugs will only show that associated nematodes can be found on slugs, not that they are specifically associated with slugs. *P. pelhami* n. sp. and *P. pellio* were both found to be associated with slugs as well as earthworms [4-6] (D. Howe & D. Denver, pers. comm.), suggesting that a broader range of possible hosts needs to be surveyed.

- *P. pellio*—the type species of *Pellioditis*—is embedded well within the *Pellioditis* (syn. *Phasmarhabditis*) clade. This placement validates the synonymization of *Phasmarhabditis* with *Pellioditis*, and the valid name of this clade is unquestionably *Pellioditis*.
- The MP phylogeny allows an estimate of the **stemspecies pattern**:

  Body cylindrical, robust, lateral field with three prominent ridges, lips in three pairs, one dorsal and two subventral, stoma triangular, buccal cavity three-edged prismatic, relatively short, stegostom to about half the stoma length, glottoid apparatus well developed, each sector with warts, pharynx with weak median bulb, gonochoristic, female amphidelphic, vulva a transverse slit in midbody, at first ovi- and later ovoviviparous, female tail conical-shaped, phasmids papilliform projecting at a point midway between anus and tail terminus, testis ventrally reflexed, male bursa well developed, peloderan, anteriorly open, nine pairs of bursal papillae (GP), three pairs anterior of the cloaca, two adcloacal, formula v1,v2,v3/(v4,ad)/v5(v6,v7,pd)ph, GP1 on level of spiculum head, GP1 to GP3 evenly spaced, GP4 and GP5 very close, GP6 separate, phasmids (ph) papilliform, spicules separate, nearly straight, like a blade, head not offset, gubernaculum spatula-shaped, following the contour of spicules to half of their length, one precloacal sensillum inconspicuous and two postcloacal sensilla conspicuous. Necromenic in gastropods (and possibly earthworms).

Conclusion

Here, we present a description of a species, *P. pelhami* n. sp., which has been used in previous

comparative developmental and systematics studies [3, 4, 6, 13-17, 26, 37-47, 49-51, 53-56, 97] and in investigations of its pathogenicity to gastropods and earthworms [7]. We also present a new comprehensive redescription of *P. pellio* including sequence data, which we use to include *P. pellio* in a phylogenetic framework for related species (Fig 10).

Our new phylogenetic results clearly show that *P. pellio* is embedded in a monophyletic group containing all species described as *Phasmarhabditis*. Because of the confusing history of the taxonomy of this group (see S1 File), because *P. pellio* was never included in *Phasmarhabditis*, and probably because it was originally described from earthworms and not slugs, *P. pellio* has been systematically omitted from consideration in descriptions of new *"Phasmarhabditis"* species. As a consequence, a recently described species, *P. bohemica*, is synonymous with *P. pellio*, as shown here. There is no more ambiguity about the validity of synonymizing *Phasmarhabditis* with *Pellioditis*. Since *P. pellio* is the senior name-bearing type species, *Pellioditis* is the name of the clade, and the name *Phasmarhabditis* must be retired (see also S1 File).

Fortunately, DNA sequences are easily obtained from rhabditid nematodes, allowing their use as barcodes to help identify species as well as phylogenetically informative characters that can place species within a taxon, identify sister taxa and provide a framework for evolutionary analyses. As we and others have found, the rapidly evolving ITS1 and ITS2 regions from the nuclear ribosomal RNA gene cluster are easily amplified by PCR and make excellent barcodes [22]. Nearly complete sequences for the 18S and 28S ribosomal RNA genes also provide excellent phylogenetic information for a range of phylogenetic relationships in Rhabditidae and other nematodes, often yielding phylogenetic results very consistent with whole-genome studies, although more genes tend to improve resolution [17, 100, 101]. At a minimum, we encourage researchers to sequence as much of the ribosomal RNA gene cluster as possible. Another excellent resource is the existence of living collections, such as the cryogenic archives at the *Caenorhabditis* Genetics Center, which accepts not only *Caenorhabditis* but other rhabditid species as well, including the *Pellioditis* species described here. Living strains allow researchers to compare features that cannot be easily compared with static descriptions and slides such as behavior and development [43], and

allow tests for reproductive isolation and pathogenesis of potential hosts [7, 22].

According to the more highly resolved MP cladogram (Fig 10A), *Angiostoma norvegicus* appears to branch off within *Pellioditis,* rendering the taxon paraphyletic. Because all *Angiostoma* sequences for the genes used here are very similar and different from sequences of other species, we are confident that these data represent the Angiostomatidae clade. This cladogram thus places Angiostomatidae far apart from *Agfa*, which is the sister group of *Pellioditis*. If this phylogenetic hypothesis holds true, obligatory gastropod parasitism evolved twice independently within Rhabditidae. The closest relative of Angiostomatidae among *Pellioditis* is currently unknown, although our MP phylogeny suggests that it might be a species of the *Pellio* group. More molecular and ecological data are needed to more confidently resolve these relationships and shed light on the evolutionary path from necromeny or gastropod pathogenicity to parasitism.

We found that earthworm-associated *P. pellio* and *P. pelhami* n. sp. belong to two clearly distinct groups within *Pellioditis.* Thus, the exploitation of earthworms as hosts could have evolved twice independently. Given that both species were also found to be associated with gastropods, and at least *P. pelhami* n. sp. was shown to be pathogenic to gastropods and earthworms, it is also possible that an association with both host taxa is an ancestral feature of the clade. Whether any of the other *Pellioditis* species can be found on earthworms is unknown since most of them were isolated in studies that specifically investigated only gastropods. Thus, a more thorough survey of soil invertebrates for the presence of *Pellioditis* nematodes is warranted to obtain a better picture of the host range and biology of these species. In this context, It is relevant that *P. pellio* [6] and *P. akhaldaba* [73] were also found in *Galleria melonella* larvae that were used as baits for entomopathogenic nematodes. Although it is most likely that this observation is due to an opportunistic colonization of a decaying corpse, it cannot be ruled out that these species can naturally infect live insect larvae.

It is desirable to have the most comprehensive knowledge possible of the ecology and biology of a species that is developed as a biological control agent before it is released. *P. hermaphrodita* has been tested for non-target effects on several invertebrate taxa and does not appear to affect the earthworms tested [102]. However, even this species is not without concern, since a recent study [4] found Nemaslug®-

derived strains of *P. hermaphrodita* in California, Oregon and New Zealand, places where Nemaslug® is not licensed for biocontrol. Going forward, more data on ecology and natural or potential host ranges are needed for *Pellioditis* species.

References

1. Heimpel GE, Mills NJ. Negative consequences of biological control. Biological Control: Ecology and Applications. Cambridge, England: Cambridge University Press; 2017. p. 83-113.
2. Sudhaus W. An update of the catalogue of paraphyletic 'Rhabditidae' (Nematoda) after eleven years. Soil Organisms. 2023;95(1):95-116. doi: 10.25674/so95iss1id312.
3. Fitch DHA, Baird SE, Emmons SW. Four worms of a different color. Worm Breeder's Gazette. 1992;12(2):14.
4. Howe DK, Ha AD, Colton A, De Ley IT, Rae RG, Ross J, et al. Phylogenetic evidence for the invasion of a commercialized European *Phasmarhabditis hermaphrodita* lineage into North America and New Zealand. PLoS One. 2020;15(8):e0237249. Epub 2020/08/18. doi: 10.1371/journal.pone.0237249. PubMed PMID: 32804938; PubMed Central PMCID: PMC7430733.
5. Maupas E. Essais d'hybridation chez des nématodes. Bulletin Biologique de la France et de la Belgique. 1919;52(3):466-98.
6. Nermuťʼ J, Půža V, Mekete T, Mráček Z. *Phasmarhabditis bohemica* n. sp. (Nematoda: Rhabditidae), a slug-parasitic nematode from the Czech Republic. Nematology. 2017;19(1):93-107. doi: 10.1163/15685411-00003034.
7. Leung N, Fitch D, Paine T, Tandingan De Ley I. Pathogenicity of two isolates of a new *Phasmarhabditis* (Nematoda) species in earthworms and invasive slugs. IOBC-WPRS Bulletin. 2020;150:147-51.
8. Sudhaus W. Preadaptive plateau in Rhabditida (Nematoda) allowed the repeated evolution of zooparasites, with an outlook on evolution of life cycles within Spiroascarida. Palaeodiversity. 2010;3(Supplement):113-78.
9. Hertwig P. Beobachtungen über die Fortpflanzungsweise und die systematische Einteilung der Regenwurmnematoden. Zeitschrift für Wissenschaftliche Zoologie. 1922;119(4):539-58.
10. Sudhaus W. Dispersion of nematodes (Rhabditida) in the guts of slugs and snails. Soil Organisms. 2018;90(3):101-14. doi: 10.25674/4jp6-0v30.

11. Sudhaus W. Phylogenetic systematisation and catalogue of paraphyletic "Rhabditidae" (Secernentea, Nematoda). Journal of Nematode Morphology and Systematics. 2011;14(2):113-78.

12. ICZN. International Code of Zoological Nomenclature. 4th ed. ed. London, UK: The International Trust for Zoological Nomenclature; 1999. 306 p.

13. Huang RE, Ye W, Ren X, Zhao Z. Morphological and molecular characterization of *Phasmarhabditis huizhouensis* sp. nov. (Nematoda: Rhabditidae), a new rhabditid nematode from South China. PLoS One. 2015;10(12):e0144386. Epub 2015/12/18. doi: 10.1371/journal.pone.0144386. PubMed PMID: 26674768; PubMed Central PMCID: PMC4686017.

14. Pieterse A, Malan AP, Ross JL. Nematodes that associate with terrestrial molluscs as definitive hosts, including *Phasmarhabditis hermaphrodita* (Rhabditida: Rhabditidae) and its development as a biological molluscicide. Journal of Helminthology. 2017;91(5):517-27. doi: 10.1017/S0022149x16000572.

15. Nermuť J, Půža V, Mekete T, Mráček Z. *Phasmarhabditis bonaquaense* n. sp. (Nematoda: Rhabditidae), a new slug-parasitic nematode from the Czech Republic. Zootaxa. 2016;4179(3):530-46. Epub 2016/11/05. doi: 10.11646/zootaxa.4179.3.8. PubMed PMID: 27811685.

16. Nermuť J, Půža V, Mráček Z. *Phasmarhabditis apuliae* n. sp. (Nematoda: Rhabditidae), a new rhabditid nematode from milacid slugs. Nematology. 2016;18:1095-112. doi: 10.1163/15685411-00003017.

17. Kiontke K, Barrière A, Kolotuev I, Podbilewicz B, Sommer R, Fitch DHA, et al. Trends, stasis, and drift in the evolution of nematode vulva development. Current Biology. 2007;17(22):1925-37. Epub 2007/11/21. doi: 10.1016/j.cub.2007.10.061. PubMed PMID: 18024125.

18. Schneider A. Monographie der Nematoden. Berlin, Germany: Georg Reimer; 1866. 357 p.

19. Fitch DHA, Bugaj-Gaweda B, Emmons SW. 18S ribosomal RNA gene phylogeny for some Rhabditidae related to *Caenorhabditis*. Mol Biol Evol. 1995;12(2):346-58. Epub 1995/03/01. doi: 10.1093/oxfordjournals.molbev.a040207. PubMed PMID: 7700158.

20. Kiontke K, Fitch DH. The phylogenetic relationships of *Caenorhabditis* and other rhabditids. WormBook. 2005:1-11. Epub 2007/12/01. doi: 10.1895/wormbook.1.11.1. PubMed PMID: 18050394; PubMed Central PMCID: PMC4781183.

21. Kiontke K, Gavin NP, Raynes Y, Roehrig C, Piano F, Fitch DH. *Caenorhabditis* phylogeny predicts convergence of hermaphroditism and extensive intron loss. Proc Natl Acad Sci U S A. 2004;101(24):9003-8.

Epub 2004/06/09. doi: 10.1073/pnas.0403094101. PubMed PMID: 15184656; PubMed Central PMCID: PMC428462.

22. Kiontke KC, Felix MA, Ailion M, Rockman MV, Braendle C, Penigault JB, et al. A phylogeny and molecular barcodes for *Caenorhabditis*, with numerous new species from rotting fruits. BMC Evol Biol. 2011;11:339. Epub 2011/11/23. doi: 10.1186/1471-2148-11-339. PubMed PMID: 22103856; PubMed Central PMCID: PMC3277298.

23. Sudhaus W, Fitch DHA. Comparative studies on the phylogeny and systematics of the Rhabditidae (Nematoda). Journal of Nematology. 2001;33(1):1-70. PubMed PMID: 19265873; PubMed Central PMCID: PMC2620500.

24. De Grisse AT. Redescription ou modifications de quelques techniques utilisées dans l'étude des nématodes phytoparasitaires. Mededelingen Rijksfakulteit Landbouwwetenschappen Gent. 1969;34:351-69.

25. Seinhorst JW. A rapid method for the transfer of nematodes from fixative to anhydrous glycerin. Nematologica. 1959;4(1):67-9. doi: 10.1163/187529259X00381.

26. Tandingan De Ley I, Mc Donnell R, Lopez S, Paine TD, De Ley P. *Pellioditis hermaphrodita* (Nematoda: Rhabditidae), a potential biocontrol agent isolated for the first time from invasive slugs in North America. Nematology. 2014;16:1129-38. Epub Nov. 19, 2014. doi: 10.1163/15685411-00002838.

27. Fitch DHA, Emmons SW. Variable cell positions and cell contacts underlie morphological evolution of the rays in the male tails of nematodes related to *Caenorhabditis elegans*. Dev Biol. 1995;170(2):564-82. Epub 1995/08/01. doi: 10.1006/dbio.1995.1237. PubMed PMID: 7649384.

28. Sudhaus W. Order Rhabditina: "Rhabditidae". In: Schmidt-Rhaesa A, editor. Hbk Zool. Handbook of Zoology. Berlin, Germany: W. de Gruyter; 2014. p. 537-55.

29. Sudhaus W, Fürst von Lieven A. A phylogenetic classification and catalogue of the Diplogastridae (Secernentea, Nematoda). Journal of Nematode Morphology and Systematics. 2003;6(1):43-89.

30. Thompson JD, Gibson TJ, Plewniak F, Jeanmougin F, Higgins DG. The CLUSTAL_X windows interface: flexible strategies for multiple sequence alignment aided by quality analysis tools. Nucleic Acids Research. 1997;25(24):4876-82. doi: 10.1093/nar/25.24.4876.

31. Maddison WP, Maddison DR. Mesquite: a modular system for evolutionary analysis. 3.70 ed2021.

32. Wiens JJ, Morrill MC. Missing data in phylogenetic analysis: reconciling results from simulations and empirical data. Syst Biol. 2011;60(5):719-31. Epub 2011/03/31. doi: 10.1093/sysbio/syr025. PubMed PMID: 21447483.

33. Swofford DL. PAUP*. Phylogenetic analysis using parsimony (*and other methods). 4a.168 ed. Sunderland, MA, USA: Sinauer Associates; 2003.

34. Hillis DM, Huelsenbeck JP, Cunningham CW. Application and accuracy of molecular phylogenies. Science. 1994;264(5159):671-7. Epub 1994/04/29. doi: 10.1126/science.8171318. PubMed PMID: 8171318.

35. Stamatakis A. RAxML version 8: a tool for phylogenetic analysis and post-analysis of large phylogenies. Bioinformatics. 2014;30(9):1312-3. Epub 2014/01/24. doi: 10.1093/bioinformatics/btu033. PubMed PMID: 24451623; PubMed Central PMCID: PMC3998144.

36. Creevey CJ, McInerney JO. Clann: investigating phylogenetic information through supertree analyses. Bioinformatics. 2005;21(3):390-2. Epub 2004/09/18. doi: 10.1093/bioinformatics/bti020. PubMed PMID: 15374874.

37. Duerr JS, Han HP, Fields SD, Rand JB. Identification of major classes of cholinergic neurons in the nematode *Caenorhabditis elegans*. J Comp Neurol. 2008;506(3):398-408. Epub 2007/11/29. doi: 10.1002/cne.21551. PubMed PMID: 18041778.

38. Azevedo RBR, Cunha A, Emmons SW, Leroi AM. The demise of the Platonic worm. Nematology. 2000;2:71-9. doi: 10.1163/156854100508917.

39. Cunha A, Azevedo RBR, Emmons SW, Leroi AM. Developmental biology: variable cell number in nematodes. Nature. 1999;402(6759):253. doi: 10.1038/46211.

40. Flemming AJ, Shen ZZ, Cunha A, Emmons SW, Leroi AM. Somatic polyploidization and cellular proliferation drive body size evolution in nematodes. Proc Natl Acad Sci U S A. 2000;97(10):5285-90. Epub 2000/05/11. doi: 10.1073/pnas.97.10.5285. PubMed PMID: 10805788; PubMed Central PMCID: PMC25820.

41. LaMunyon CW, Ward S. Evolution of sperm size in nematodes: sperm competition favours larger sperm. Proc Biol Sci. 1999;266(1416):263-7. Epub 1999/03/19. doi: 10.1098/rspb.1999.0631. PubMed PMID: 10081163; PubMed Central PMCID: PMC1689669.

42. So S, Garan Y, Miyahara K, Ohshima Y. Body size change in various nematodes depending on bacterial food, sex and growth temperature. Worm. 2012;1(2):93-7. doi: 10.4161/worm.20175. PubMed PMID: 24058830; PubMed Central PMCID: PMC3670232.

43. Brauchle M, Kiontke K, MacMenamin P, Fitch DH, Piano F. Evolution of early embryogenesis in rhabditid nematodes. Dev Biol. 2009;335(1):253-62. Epub 2009/08/01. doi: 10.1016/j.ydbio.2009.07.033. PubMed PMID: 19643102; PubMed Central PMCID: PMC2763944.

44. Ivanova E, Clausi M, Sparacio I, Spiridonovi S. Preliminary data on the parasite survey of terrestrial gastropods of Sicily. Russ J Nematol. 2019;27(1):37-45. doi: 10.24411/0869-6918-2019-10005.

45. Ivanova ES, Geraskina AP, Spiridonov SE. Two new species of *Phasmarhabditis* Andrassy, 1976 (Nematoda: Rhabditidae) associated with land snails in Northwest Caucasus, Russian Federation: description and molecular affiliation. Nematology. 2020;22(2):179-97. doi: 10.1163/15685411-00003299.

46. Ivanova ES, Spiridonov SE. *Phasmarhabditis quinamensis* sp. n. (Nematoda: Rhabditidae) from tropical terrestrial gastropods in southern Vietnam. Nematology. 2022;24(2):225-39. doi: 10.1163/15685411-bja10126.

47. Pieterse A, Rowson B, Tiedt L, Malan AP, Haukeland S, Ross JL. *Phasmarhabditis kenyaensis* n. sp. (Nematoda: Rhabditidae) from the slug, *Polytoxon robustum*, in Kenya. Nematology. 2021;23(2):229-45. doi: 10.1163/15685411-bja10040.

48. Pieterse A, Tiedt LR, Malan AP, Ross JL. First record of *Phasmarhabditis papillosa* (Nematoda: Rhabditidae) in South Africa, and its virulence against the invasive slug, *Deroceras panormitanum*. Nematology. 2017;19(9):1035-50. doi: 10.1163/15685411-00003105.

49. Rae R. *Phasmarhabditis hermaphrodita*—a new model to study the genetic evolution of parasitism. Nematology. 2017;19(4):375-87. doi: 10.1163/15685411-00003053.

50. Ross JL, Ivanova ES, Hatteland BA, Brurberg MB, Haukeland S. Survey of nematodes associated with terrestrial slugs in Norway. J Helminthol. 2016;90(5):583-7. Epub 2015/09/29. doi: 10.1017/S0022149X15000784. PubMed PMID: 26411747.

51. Ross JL, Ivanova ES, Sirgel WF, Malan AP, Wilson MJ. Diversity and distribution of nematodes associated with terrestrial slugs in the Western Cape Province of South Africa. J Helminthol. 2012;86(2):215-21. Epub 2011/07/07. doi: 10.1017/S0022149X11000277. PubMed PMID: 21729386.

52. Ross JL, Pieterse A, Malan AP, Ivanova E. *Phasmarhabditis safricana* n. sp. (Nematoda: Rhabditidae), a parasite of the slug *Deroceras reticulatum* from South Africa. Zootaxa. 2018;4420(3):391-404. Epub 2018/10/14. doi: 10.11646/zootaxa.4420.3.5. PubMed PMID: 30313534.

53. Tandingan De Ley I, Holovachov O, Mc Donnell RJ, Bert W, Paine TD, De Ley P. Description of *Phasmarhabditis californica* n. sp. and first report of *P. papillosa* (Nematoda: Rhabditidae) from invasive slugs in the USA. Nematology. 2016;18(2):175-93. Epub Mar. 18, 2016. doi: 10.1163/15685411-00002952.

54. Zhang C, Liu J, Xu M, Sun J, Yang S, An X, et al. *Heterorhabditidoides chongmingensis* gen. nov., sp. nov. (Rhabditida: Rhabditidae), a novel member of the entomopathogenic nematodes. J Invertebr Pathol. 2008;98(2):153-68. Epub 2008/04/16. doi: 10.1016/j.jip.2008.02.011. PubMed PMID: 18410943.

55. Gorgadze O, Troccoli A, Fanelli E, Tarasco E, De Luca F. *Phasmarhabditis thesamica* n. sp. (Nematoda: Rhabditidae), a new slug nematode from southern slope of Caucasus, Georgia. Nematology. 2022;24(6):617-29. doi: 10.1163/15685411-bja10154.

56. Waki T, Hino A, Umeda K. *Angiostoma namekuji* n. sp. (Nematoda: Angiostomatidae) from terrestrial slugs on Oshiba Island in the Seto Inland Sea, Japan. Syst Parasitol. 2018;95(8-9):913-20. doi: 10.1007/s11230-018-9824-y.

57. Fitch DHA. Evolution of "Rhabditidae" and the male tail. J Nematol. 2000;32(3):235-44. Epub 2000/09/01. PubMed PMID: 19270972; PubMed Central PMCID: PMC2620451.

58. Fitch DHA. Evolution of male tail development in rhabditid nematodes related to *Caenorhabditis elegans*. Syst Biol. 1997;46(1):145-79. Epub 1997/03/01. doi: 10.1093/sysbio/46.1.145. PubMed PMID: 11975351.

59. De Ley P, Vandevelde MC, Mounport D, Baujard P, Coomans A. Ultrastructure of the stoma in Cephalobidae, Panagrolaimidae and Rhabditidae, with a proposal for a revised stoma terminology in Rhabditida (Nematoda). Nematologica. 1995;41(2):153-82. doi: 10.1163/003925995x00143.

60. Rashid F, Geraert E, Coomans A, Suatmadji W. Cephalobidae from the Krakatau Region (Nematoda, Rhabditida). Nematologica. 1988;34(2):125-43. doi: 10.1163/002825988x00224.

61. Hooper DJ, Wilson MJ, Rowe JA, Glen DM. Some observations on the morphology and protein profiles of the slug-parasitic nematodes *Phasmarhabditis hermaphrodita* and *P. neopapillosa* (Nematoda: Rhabditidae). Nematology. 1999;1:173-82. doi: 10.1163/156854199508144.

62. Poinar GO. Associations between nematodes (Nematoda) and oligochaetes (Annelida). P Helm Soc Wash. 1978;45(2):202-10.

63. Keilin D. Parasitic autotomy of the host as a mode of liberation of coelomic parasites from the body of the earthworm. Parasitology. 1925;17(2):170-2. Epub Apr 6, 2009. doi: 10.1017/S0031182000004510.

64. Maupas E. La mue et l'enkystement chez les nématodes. Archives de Zoologie Expérimentale et Générale. 1899;Series 3, Volume 27(7):563-628 (with plates XVI-XVIII).
65. Somers JA, Shorey HH, Gaston LK. Reproductive-biology and behavior of *Rhabditis pellio* (Schneider) (Rhabditida-Rhabditidae). Journal of Nematology. 1977;9(2):143-8. PubMed PMID: 19305581; PubMed Central PMCID: PMC2620229.
66. Örley L. Die Rhabditiden und ihre medicinische Bedeutung. Berlin: R. Friedländer & Sohn; 1886 1886. 84 p.
67. Reiter M. Zur Systematik und Ökologie der zweigeschlechtlichen Rhabditiden. Arbeiten aus dem Zoologischen Institut der Universität Innsbruck. 1928;3(4):3-94.
68. Völk J. Die Nematoden der Regenwürmer und aasbesuchenden Käfer. Zoologische Jahrbücher: Abteilung für Systematik, Ökologie und Geographie der Tiere. 1950;79(1/2):1-70.
69. Bütschli O. Beiträge zur Kenntniss der freilebenden Nematoden. Nova Acta der Ksl Leop-Carol Deutschen Akademie der Naturforscher. 1873;36(5):1–144.
70. Thorne G. Principles of nematology. New York, NY, USA: McGraw-Hill; 1961. 553 p.
71. Aubertot M. Nématodes d'Alsace. Observations sur le *Rhabditis pellio* Schneider. Bulletin de l'Association Philomathique d'Alsace et de Lorraine. 1927;7(3):178-90.
72. Stock SP, Camino NB. *Pellioditis pellio* (Schneider) (Nematoda, Rhabditidae) parasitizing *Scaptia (Scaptia) lata* (Guerin-Meneville) (Diptera, Tabanidae). Mem I Oswaldo Cruz. 1991;86(2):219-22. doi: 10.1590/S0074-02761991000200011.
73. Ivanova ES, Gorgadze OA, Lortkhipanidze MA, Spiridonov SE. *Phasmarhabditis akhaldaba* sp. n. associated with a slug *Deroceras reticulatum* in Lesser Caucasus mountains in Republic of Georgia. Russ J Nematol. 2021;29(1):75-88. doi: 10.24411/0869-6918-2021-10008.
74. Azzam KM. Description of the nematode *Phasmarhabditis tawfiki* n. sp. isolated from Egyptian terrestrial snails and slugs. J Egypt Ger Soc Zool. 2003;(42D): Invertebrate Zoology & Parasitology:79-87.
75. Sudhaus W. Various evolutionary avenues of Nematoda to parasitism in Gastropoda. Soil Organisms. 2018;90(3):115-22. Epub Dec. 1, 2018. doi: 10.25674/1yds-c108.
76. Sudhaus W. Vergleichende Untersuchungen zur Phylogenie, Systematik, Ökologie, Biologie und Ethologie der Rhabditidae (Nematoda). Zoologica. 1976;43(125):1-229.

77. Dougherty EC, Calhoun HG. Experiences in culturing *Rhabditis pellio* (Schneider, 1866) Bütschli, 1873 (Nematoda, Rhabditidae), and related soil nematodes. P Helm Soc Wash. 1948;15(2):55-68.

78. Camino NB, Stock PS. Ciclo de vida de *Pellioditis pellio* (Schneider) (Nematoda: Rhabditidae) parasito de larvas de *Scaptia (Scaptia) lata* (Guerin-Meneville) (Diptera: Tabanidae). Acta Ent Chilena. 1993;18:73-5.

79. Poinar GO, Thomas GM. *Rhabditis pellio* Schneider (Nematoda) from the earthworm, *Aporrectodea trapezoides* Duges (Annelida). Journal of Nematology. 1975;7(4):374-9. PubMed PMID: 19308185.

80. Eveland LK, Fujino T, Fried B. Scanning electron microscopical observations of *Pellioditis pellio* (Nematoda, Secernentia). T Am Microsc Soc. 1990;109(1):84-90.

81. Goodchild CG, Irwin GH. Occurrence of nematodes *Rhabditis anomala* and *R. pellio* in oligochaetes *Lumbricus rubellus* and *L. terrestris*. T Am Microsc Soc. 1971;90(2):231-7. doi: 10.2307/3225031.

82. Zaborski ER, Soeken Gittenger LA, Roberts SJ. A possible *Phasmarhabditis* sp. (Nematoda: Rhabditidae) isolated from *Lumbricus terrestris* (Oligochaeta: Lumbricidae). Journal of Invertebrate Pathology. 2001;77(4):284-7. Epub 2001/07/05. doi: 10.1006/jipa.2001.5024. PubMed PMID: 11437532.

83. Dougherty EC. The genera of the subfamily Rhabditinae Micoletzky, 1922 (Nematoda). In: Dayal JS, Kunwar Suresh, editor. Thapar Commemoration Volume 1953 A Collection of Articles Presented to Prof G S Thapar on his Sixtieth Birthday. Lucknow, Uttar Pradesh, India: Department of Zoology, University of Lucknow; 1953. p. 69-76.

84. Andrássy I. Evolution as a Basis for the Systematization of Nematodes. London: Pitman; 1976. 288 p.

85. Schneider A. Ueber eine Nematodenlarve und gewisse Verschiedenheiten in den Geschlechtsorganen der Nematoden. Zeitschrift für Wissenschaftliche Zoologie. 1859;10(1):176-8.

86. Shinohara T, Yokoo T. On a new *Rhabditis* species (Nematoda, Rhabditidae) found in the alimentary organs of *Incilaria confusa* (Cockerell) in Japan. Kurume Igakkai zasshi [Journal of the Kurume Medical Association]. 1958;21(12):2580-4.

87. Shinohara T. Studies on *Rhabditis* (Nematoda, Rhabditidae). Kurume Igakkai zasshi [Journal of the Kurume Medical Association]. 1960;23(7):2777-819.

88. Kreis HA. Beiträge zur Kenntnis parasitischer Nematoden. XXVI. Ein neuer Nematode aus der Schnecke *Fruticicola (Acusta) sieboldiana* Pfeifer, *Rhabditis fruticicolae* n. sp. (Rhabditidoidea; Rhabditidae). Jahrbuch - Naturhistorisches Museum der Stadt Bern. 1967;4(1963-1965):171-81.

89. Ivanova ES, Spiridonov SE. *Phasmarhabditis meridionalis* sp n. (Nematoda: Rhabditidae) from a land snail *Quantula striata* (Gastropoda: Dyakiidae) from southern Vietnam. Russ J Nematol. 2017;25(2):129-40.

90. Osche G. Systematik und Phylogenie der Gattung *Rhabditis* (Nematoda). Zoologische Jahrbücher (Systematik); Zool Jb (Syst). 1952;81(3):190-280.

91. Will F. Zur Anatomie und Naturgeschichte von *Angiostoma limacis* Duj. Archiv für Naturgeschichte Jahrgang. 1848;14(1):174-9.

92. Dujardin MF. Histoire naturelle des helminthes ou vers intestinaux. Paris, France: Librairie Encyclopédique de Roret; 1845. 654 p.

93. Tadano M. Notes on a new species of parasitic nematode, *Rhabditis ikedai* n. sp. from the slug, *Incillaria confusa* Cockarell. Dobutsugaku Zasshi [Zoological Journal]. 1950;59(12):289-91.

94. Yokoo T. On a new *Rhabditis* species (Nematoda: Rhabditidae) found in the alimentary organs of snails from Nagasaki. Agricultural Bulletin of Saga University. 1968;25:63-7.

95. Ivanova ES, Clausi M, Leone D, Spiridonov SE. *Phasmarhabditis villasmundi* sp. n. infecting land gastropods in the Nature Reserve 'Speleological Complex Villasmundo—S. Alfio' in Syracuse Province, Sicily. Nematology. 2023;25(2):169-80. doi: 10.1163/15685411-bja10212.

96. Zhang CN, Liu QZ. *Phasmarhabditis zhejiangensis* sp. nov. (Nematoda: Rhabditidae), a new rhabditid nematode from Zhejiang, China. PLoS One. 2020;15(11):e0241413. Epub 2020/11/10. doi: 10.1371/journal.pone.0241413. PubMed PMID: 33166297; PubMed Central PMCID: PMC7652337.

97. Ross JL, Ivanova ES, Spiridonov SE, Waeyenberge L, Moens M, Nicol GW, et al. Molecular phylogeny of slug-parasitic nematodes inferred from 18S rRNA gene sequences. Mol Phylogenet Evol. 2010;55(2):738-43. doi: 10.1016/J.Ympev.2010.01.026.

98. Osche G. Über die gegenwärtig ablaufende Entstehung von Zwillings- und Komplementärarten bei Rhabditiden (Nematodes). (Fötalisation und Artbildung). Zoologische Jahrbücher (Systematik); Zool Jb (Syst). 1954;82(6):618-54.

99. Ellis RE. "The persistence of memory"—Hermaphroditism in nematodes. Mol Reprod Dev. 2017;84(2):144-57. Epub 2016/06/14. doi: 10.1002/mrd.22668. PubMed PMID: 27291983.

100. Ahmed M, Roberts NG, Adediran F, Smythe AB, Kocot KM, Holovachov O. Phylogenomic analysis of the phylum Nematoda: conflicts and congruences with morphology, 18S rRNA, and mitogenomes. Front Ecol Evol. 2022;9. doi: 10.3389/fevo.2021.769565.

101. van Megen H, van den Elsen S, Holterman M, Karssen G, Mooyman P, Bongers T, et al. A phylogenetic tree of nematodes based on about 1200 full-length small subunit ribosomal DNA sequences. Nematology. 2009;11(6):927-50. doi: 10.1163/156854109x456862.

102. Rae R, Verdun C, Grewal P, Robertson JF, Wilson MJ. Biological control of terrestrial molluscs using *Phasmarhabditis hermaphrodita* — progress and prospects. Pest Manag Sci. 2007;63(12):1153-64. doi: 10.1002/ps.1424. PubMed PMID: 17912686.

Supporting information

S1 Table. Taxa and GenBank accession numbers for DNA sequences used in phylogenetic analyses. (XLSX)

S1 Fig. Schematic view of the DNA alignment supermatrix and submatrices used for supertree analyses. To conserve space, only the parsimony-informative characters (alignment positions) are depicted, with different colors depicting different nucleotide character states. Labeled boxes of different colors represent the taxon and character subsets of the full supermatrix used for individual ML bootstrap analyses. See Materials and Methods for description of datasets. (JPEG)

S1 File. History of the *Pellioditis* and *Phasmarhabditis* taxa, and how the confusion between them arose. (DOCX)

Videos. Stacks of focal planes of DIC images for different parts of EM434 and SB361:

S1 Video. EM434 adult female showing posterior spermatheca, right side view.

S2 Video. EM434 adult female stoma region, left side view.

S3 Video. EM434 adult female tail, ventral view.

S4 Video. EM434 adult female pharynx region, left side view.

S5 Video. EM434 adult male stoma region, left side view.

S6 Video. EM434 adult male tail (copulatory bursa), ventral view.

S7 Video. EM434 adult male tail, right side view.

S8 Video. EM434 adult male stoma region, dorsal view.

S9 Video. EM434 spicules (lateral views) and gubernaculum (ventral view) isolated from an adult male.

S10 Video. SB361 adult female stoma region, right side view.

S11 Video. SB361 adult female tail, right subventral view.

S12 Video. SB361 adult female anterior oviduct, left side view.

S13 Video. SB361 adult female pharynx region, left side view.

S14 Video. SB361 adult male pharynx region, ventral view.

S15 Video. SB361 adult male stoma region, ventral view.

S16 Video. SB361 adult male stoma region, right side view.

S17 Video. SB361 adult male tail, right side view.

S18 Video. SB361 adult male tail, ventral view.

S19 Video. SB361 spicules (lateral views) and gubernaculum (ventral view) isolated from an adult male.

The following sequences acquired in this study have been deposited at GenBank with the following accession numbers: OR059186 (nearly complete ribosomal RNA gene repeat unit for strain EM434) and OR059187 (nearly complete ribosomal RNA gene repeat unit for strain SB361).

Acknowledgments

The authors thank Marjolein Couvreur, University of Ghent, for scanning electron microscopic images. We thank Dana Howe and Dee Denver, Oregon State University, for communicating and allowing us to cite unpublished results. Some strains were archived at and provided by the *Caenorhabditis* Genetics Center at the University of Minnesota and the New York University Rhabditid Collection. We thank Dominick

Verschelde (Curator, Ghent University Zoology Museum), Gerrit Karssen (Curator, Wageningen Nematode Collection), and Steve Nadler (Curator, Nematode Collection, University of California, Davis) for archiving type material.

Author Contributions

Conceived and designed the experiments: ITD DHAF KK WS. Performed the experiments: ITD KK DHAF WB. Analyzed the data: ITD KK DHAF. Contributed reagents/materials/analysis tools: ITD WS WB DHAF. Wrote and edited the paper: ITD KK WB WS DHAF. Provided laboratory infrastructure: ITD DHAF WB WS.

Fig 1

Click here to access/download;Figure;Fig1.tif

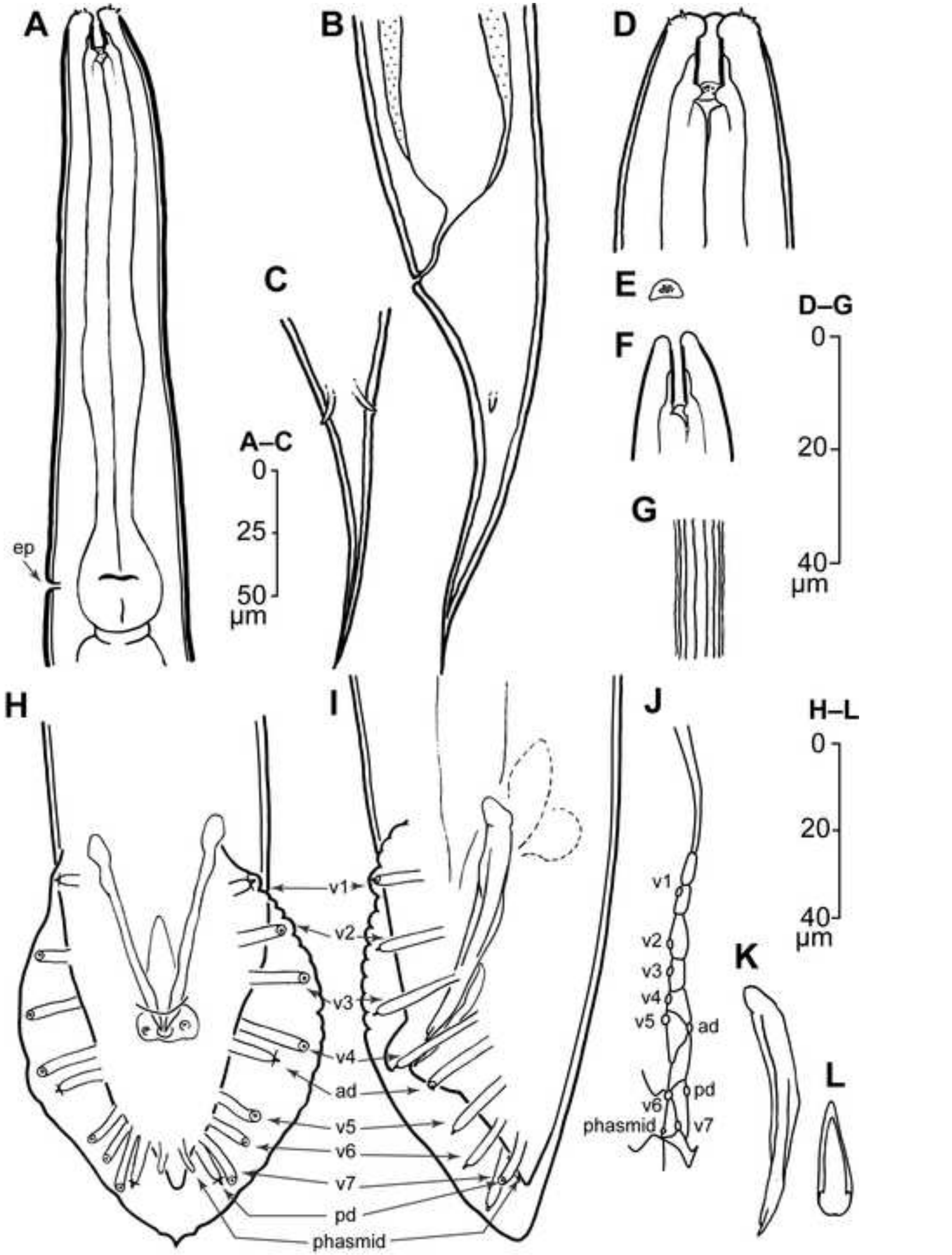

Fig 2

Click here to access/download;Figure;Fig2.tif

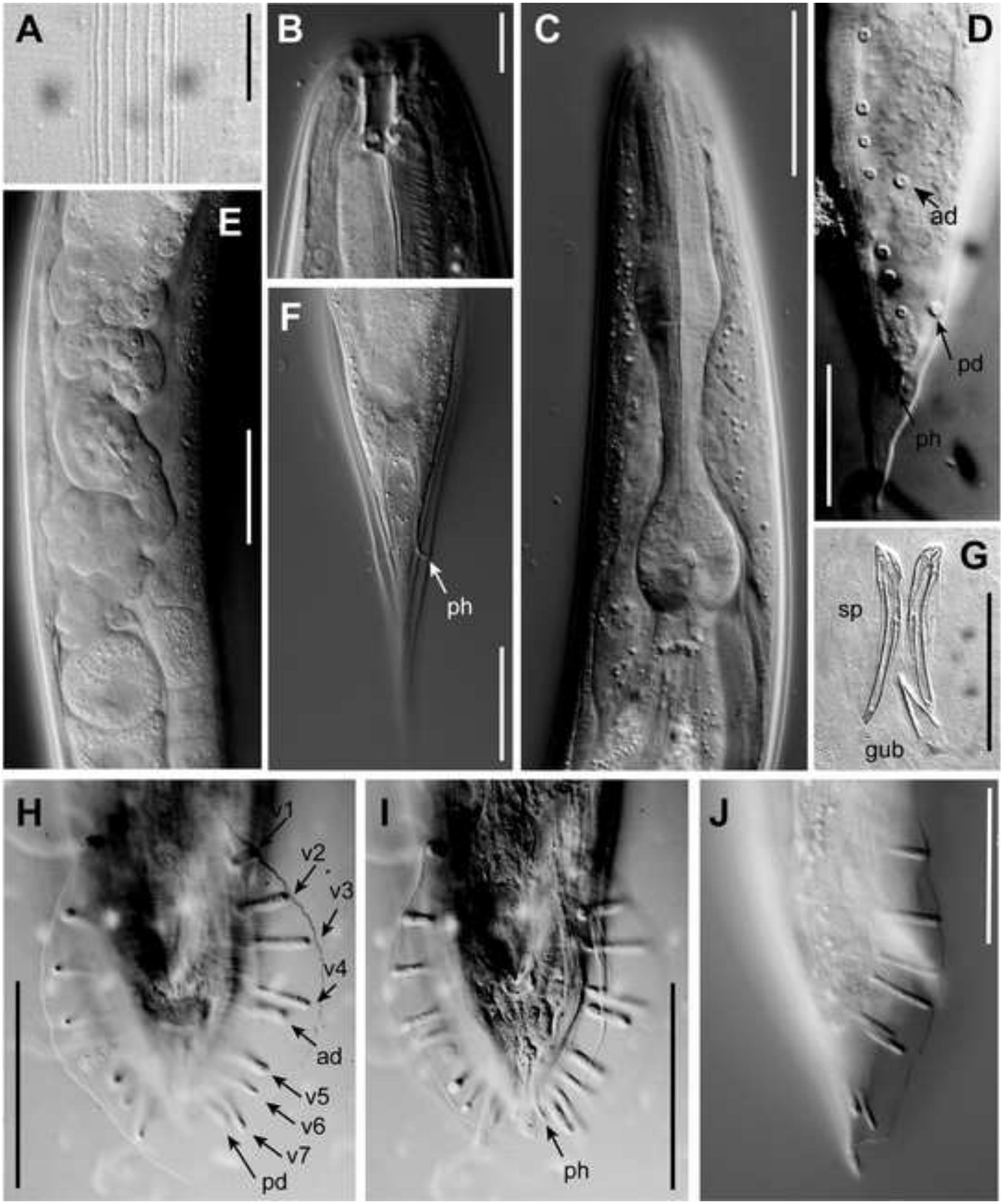

Fig 3

Click here to access/download;Figure;Fig3.tif

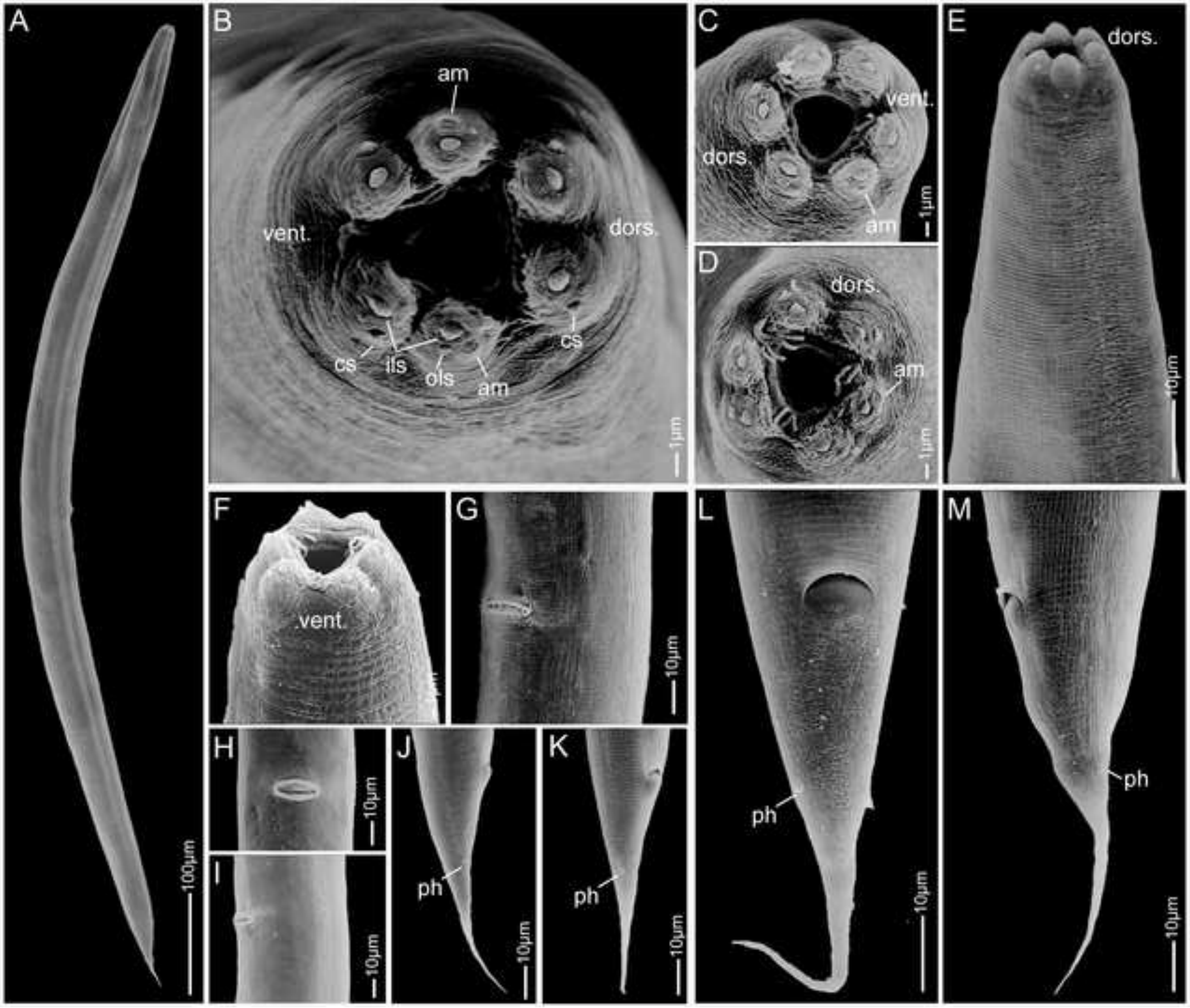

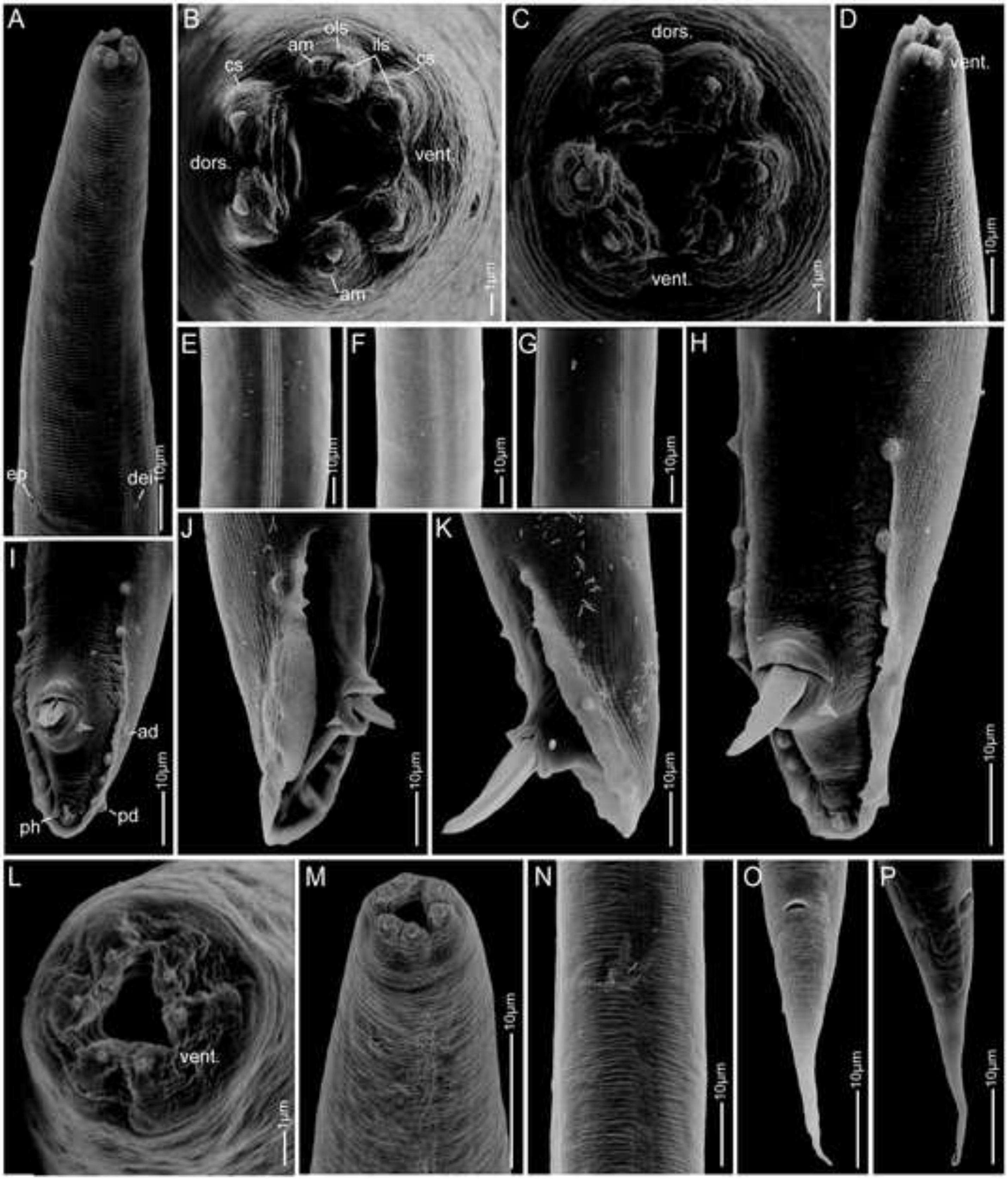
A
B
C
D
E
F
G
H
I
J
K
L
M
N
O
P
ep
dei
cs
am
ols
ils
dors.
vent.
ad
pd
ph
10µm
1µm

Fig 5

Click here to access/download;Figure;Fig5.tif

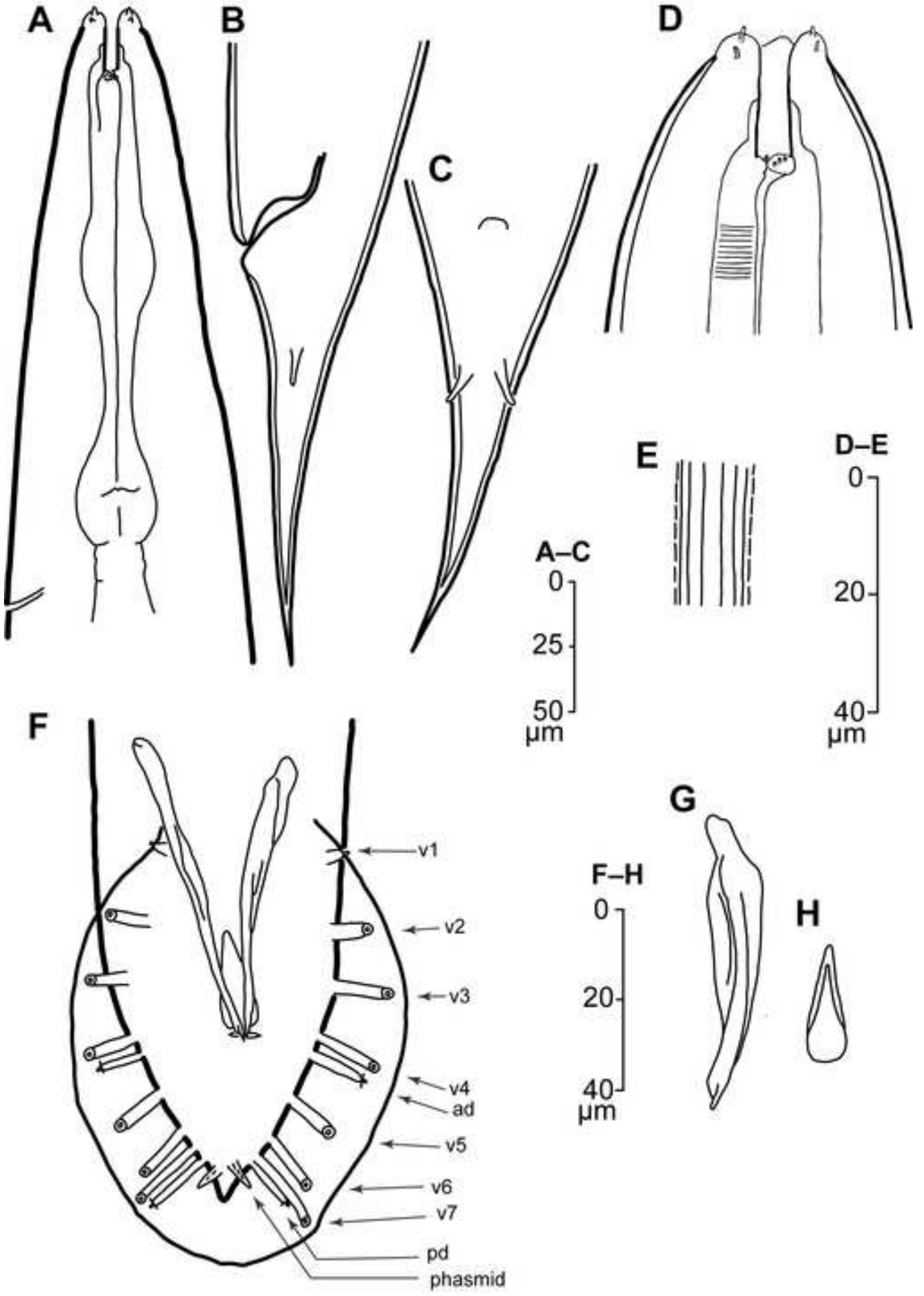

Fig 6 Click here to access/download;Figure;Fig6.tif

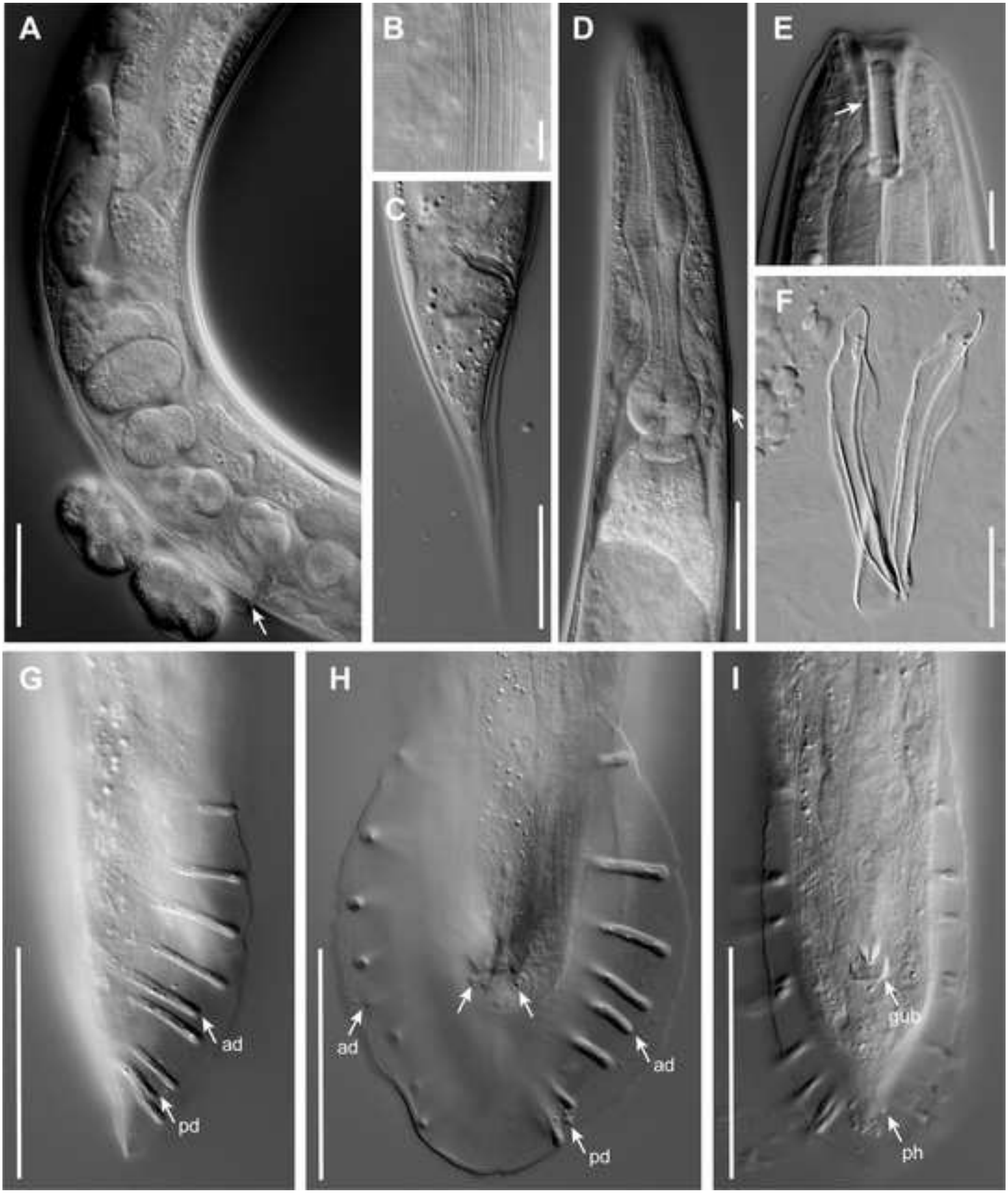

Fig 7

Click here to access/download;Figure;Fig7.tif

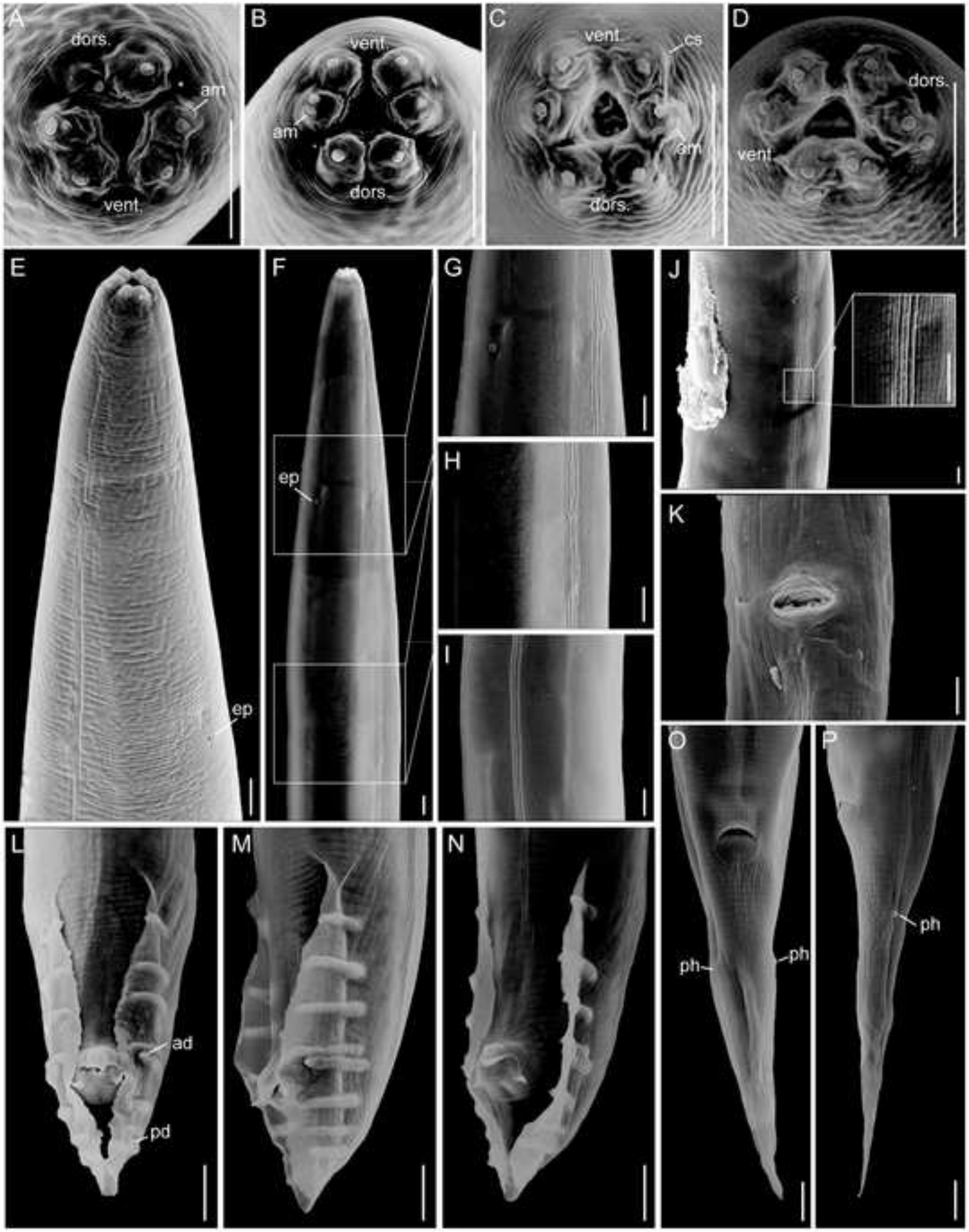

 Click here to access/download;Figure;Fig8.tif

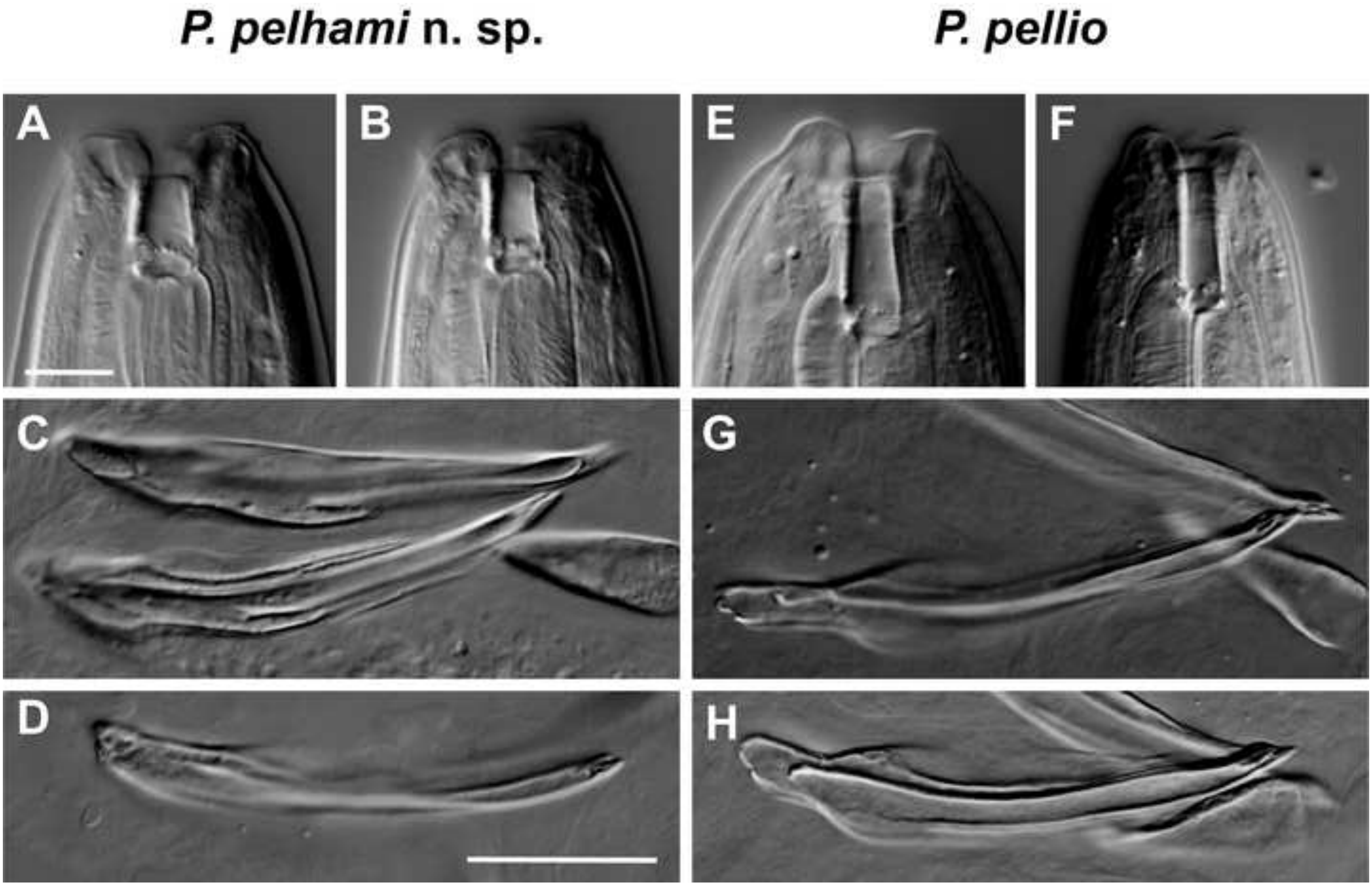

Click here to access/download;Figure;Fig9.tif

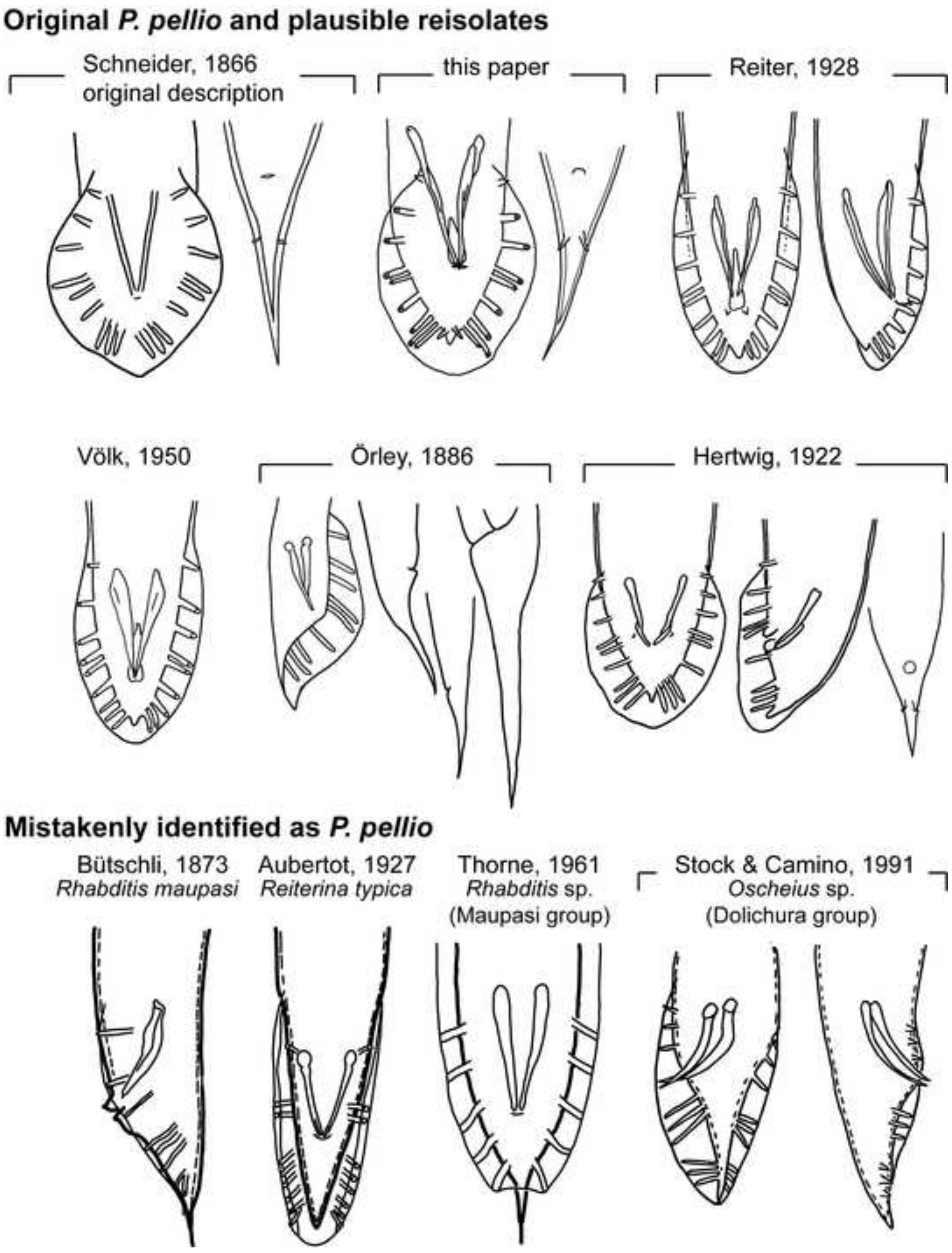

Fig 10

Click here to access/download;Figure;Fig10.eps

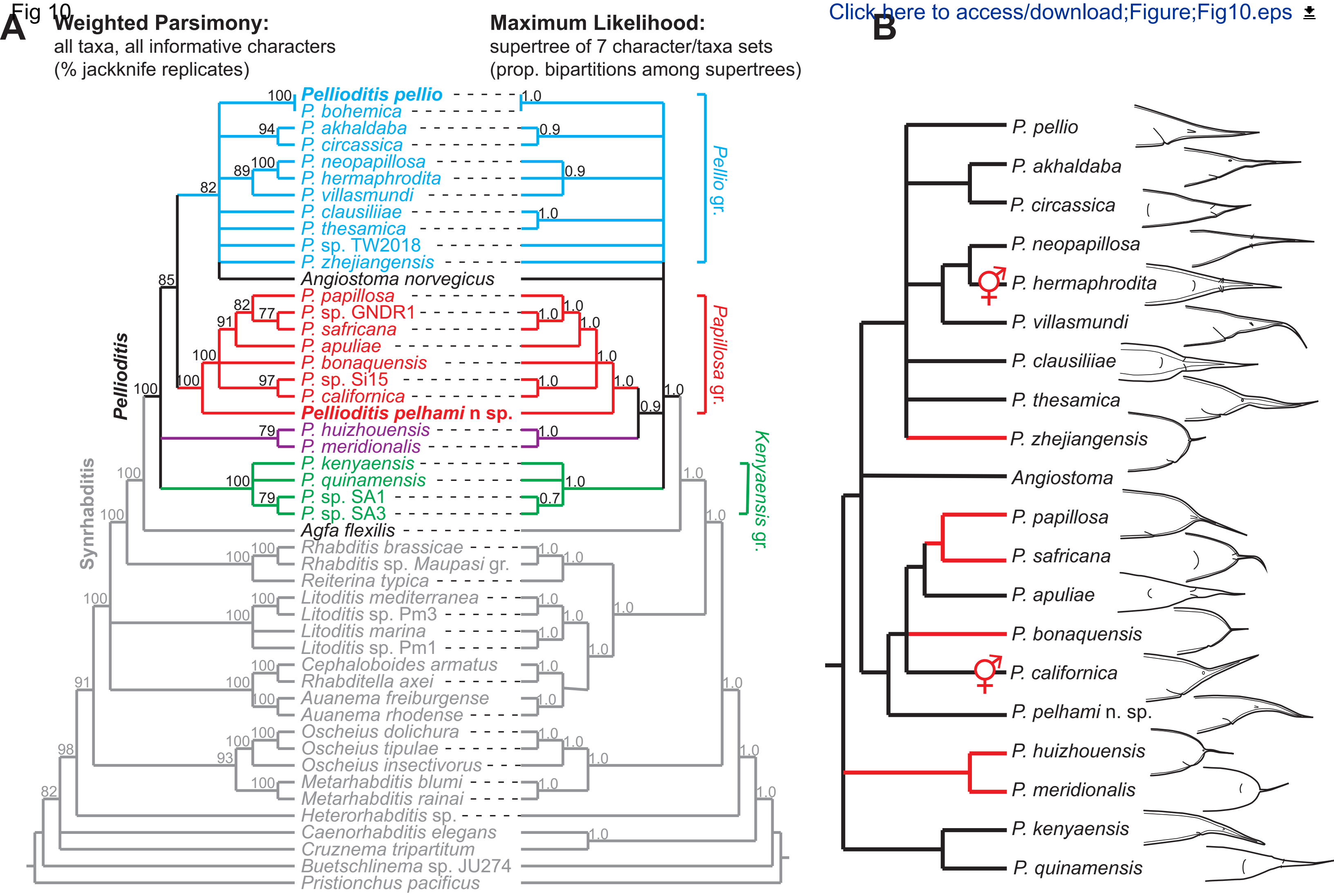

S1 Fig

Click here to access/download
Supporting Information
S1_Fig.eps

S1 Table

Click here to access/download
Supporting Information
S1_Table.xlsx

S1 File

Click here to access/download
Supporting Information
S1 _File.docx

Click here to access/download
Supporting Information - Compressed/ZIP File Archive
SupplementalVideos.zip
