# Supplementary material for: Pellioditis pelhamensis n. sp. (Nematoda: Rhabditidae) and Pellioditis pellio (Schneider, 1866), earthworm associates from different subclades within Pellioditis (syn. Phasmarhabditis Andrássy, 1976)
Source: PLoS One. 2023 Sep 6;18(9):e0288196. doi: 10.1371/journal.pone.0288196 (PMC10482300; doi:10.1371/journal.pone.0288196)
Supplement: Attachment — Submitted filename: ResponseToReviewers.pdf [file pone.0288196.s006.pdf]

New York University
<u>*A private university in the public service*</u>

David H. A. Fitch, Ph.D.
Professor of Biology
Center for Developmental Genetics, Department of Biology
New York, NY 10003
Tel.: (212) 998-8254
E-mail: david.fitch@nyu.edu

July 19, 2023

PLoS ONE

Dear Editor Aashaq Hussain Bhat:

Many thanks for the very valuable reviews of our paper, PONE-D-23-19233, re-titled "*Pellioditis pelhamensis* n. sp. (Nematoda: Rhabditidae) and *Pellioditis pellio* (Schneider, 1866), earthworm associates from different subclades within *Pellioditis* (syn. *Phasmarhabditis* Andrássy, 1976). To the best of our understanding, we have addressed all of your (Editor's) concerns, as well as those of the reviewers, as listed below:

<u>Editor's comments</u>

(Note that this letter represents the Response to Reviewers requested. Also, we have uploaded a marked-up copy of the manuscript as well as an unmarked version.)

1. I believe we have formatted our paper according to the PLOS ONE style requirements.
2. All accession numbers for data and materials (e.g. type specimens) have been assigned and are included in the manuscript.
3. We have decided not to cite "data not shown". All relevant and essential data are included in the paper, in the table, text, figures, and extensive supplemental material.
4. All guidelines for descriptions of new species have been followed. We have also registered both the relevant taxa and the paper with Zoobank and include the GUID/LSIDs in the paper (Nomenclatural Acts section).
5. We have tried to be as comprehensive as possible in reporting all the information associated with the references. (Note: we have all the references available in an EndNote "traveling library", which we can easily send to you if needed. Just let me know.)
6. We have now included the protocol for testing reproductive compatibilities in the Materials and Methods section (it was included as a footnote to a table previously, but we agree with you that it is better to include in the M&M section).
7. We have tried to fix all grammatical errors (although none were specified). I have to admit that I did not find many. I'm not sure if the editor meant the type of prose found in the species descriptions per se? We have followed the typical "telegraphic" style commonly used in most zoological descriptions. In the other parts of the text, however, I found very few such errors, but such errors (as far as I know) have now been corrected.
8. We have now written out genus names for species when they are mentioned for the first time.
9. As mentioned in note 7 above, we have used the typical format for nematode species descriptions (also as in previous nematode descriptions published in PLoS ONE, e.g. *P. huizhouensis*).

<u>Reviewer 1</u>

1. We have now provided the type specimen identifiers for the UC Davis collection. (We did not yet have them when we submitted the manuscript originally.)
2. Regarding the legend to Fig. 3G, we have removed the reference to the "lateral field", which we agree is not shown well in this figure. Instead, the lateral field is better shown in other panels. We therefore call attention to the lateral field in 3I and 4E-G.
3. We have fixed the typo regarding the species name *Angiostoma norvegicum*.

<u>Reviewer 2</u>

1. We have changed the species name from *P. pelhami* to *P. pelhamensis*, as per the reviewer's suggestion. This also entailed changing the name in all associated files and databases.
2. As per the comments made in the manuscript PDF, we have

2a. fixed the typo involving *P. hermaphrodita*,
2b. taken the reviewer's suggestion to emend *P. kenyaensis* to *P. kenyensis* (and cited the appropriate ICZN article justifying this emendation).

Request to publish the peer review history

YES, we agree to publishing the peer review history. I understand that the reviewers prefer to remain anonymous.

Many thanks for all the comments from editors and reviewers, which were all very helpful in improving the manuscript. Please let me know if there is anything else we should do. We are excited to see the paper in print as soon as possible.

Sincerely,

David H. A. Fitch
