# Supplementary material for: Pellioditis pelhamensis n. sp. (Nematoda: Rhabditidae) and Pellioditis pellio (Schneider, 1866), earthworm associates from different subclades within Pellioditis (syn. Phasmarhabditis Andrássy, 1976)
Source: PLoS One. 2023 Sep 6;18(9):e0288196. doi: 10.1371/journal.pone.0288196 (PMC10482300; doi:10.1371/journal.pone.0288196)
Supplement: Attachment — Submitted filename: ResponseToEditor.pdf [file pone.0288196.s007.pdf]

New York University
A private university in the public service

David H. A. Fitch, Ph.D.
Professor of Biology
Center for Developmental Genetics, Department of Biology
New York, NY 10003
Tel.: (212) 998-8254
E-mail: david.fitch@nyu.edu

Center for Developmental Genetics at NYU Biology

July 25, 2023

PLoS ONE

Dear Editor Aashaq Hussain Bhat:

In response to your request to revise the format of the species description, we have changed all the instances of "is" and "are", etc. that you suggested. (See copy of manuscript with Track Changes markup.) Many thanks for specifying these instances, as we were not clear about what was meant by "typical format for nematode species descriptions."

Might it be possible for PLoS ONE to specify what is meant by the phrase "typical format..." in their instructions to authors (or in a special instruction sheet for nematologists, if this is a special requirement for nematodes?). It would be very helpful to have this at the outset to avoid the efforts of going through multiple revisions. For example, the instructions could specify something like:

"For the portion of the manuscript devoted to the formal [nematode] species description, please use telegraphic style, avoiding the use of 'is', 'are', etc."

Many thanks for your diligent work on this manuscript. It is greatly appreciated!

Sincerely,

David H. A. Fitch
